# Essential oil and furanosesquiterpenes from myrrh oleo-gum resin: a breakthrough in mosquito vector management

**DOI:** 10.1007/s13659-024-00492-6

**Published:** 2025-01-20

**Authors:** Eleonora Spinozzi, Marta Ferrati, Cecilia Baldassarri, Paolo Rossi, Guido Favia, Giorgio Cameli, Giovanni Benelli, Angelo Canale, Livia De Fazi, Roman Pavela, Luana Quassinti, Cristiano Giordani, Fabrizio Araniti, Loredana Cappellacci, Riccardo Petrelli, Filippo Maggi

**Affiliations:** 1 Chemistry Interdisciplinary Project (ChIP) Research Center, School of Pharmacy, University of Camerino, Via Madonna delle Carceri, 62032 Camerino, Italy; 2 School of Biosciences and Veterinary Medicine, University of Camerino, Via Gentile III Da Varano, 62032 Camerino, Italy; 3 Department of Agriculture, Food and Environment, University of Pisa, Via del Borghetto 80, 56124 Pisa, Italy; 4 Crop Research Institute, Drnovska 507, 161 06 Prague, Czech Republic; 5 Department of Plant Biotechnology, College of Life Sciences and Biotechnology, Korea University, Seoul, 02841 Republic of Korea; 6 School of Pharmacy, University of Camerino, Camerino, Italy; 7 Instituto de Física, Universidad de Antioquia, UdeA, Calle 70 No 52-21, 050010 Medellín, Colombia; 8 Grupo Productos Naturales Marinos, Facultad de Ciencias Farmacéuticas y Alimentarias, Universidad de Antioquia, Calle 70 No. 52-21, 050010 Medellín, Colombia; 9 Dipartimento di Scienze Agrarie e Ambientali, Produzione, Territorio, Agroenergia, Università Statale di Milano, Via Celoria N. 2, 20133 Milan, Italy

**Keywords:** Arbovirus vector, *Commiphora myrrha*, *Aedes aegypti*, *Anopheles* spp., Bioinsecticide

## Abstract

**Abstract:**

Mosquitoes (Diptera: Culicidae) are vectors of various pathogens of public health concern and replacing conventional insecticides remains a challenge. In this regard, natural products represent valuable sources of potential insecticidal compounds, thus increasingly attracting research interest. *Commiphora myrrha* (T.Nees) Engl. (Burseraceae) is a medicinal plant whose oleo-gum resin is used in food, cosmetics, fragrances, and pharmaceuticals. Herein, the larvicidal potential of its essential oil (EO) was assessed on four mosquito species (*Aedes albopictus* Skuse, *Aedes aegypti* L., *Anopheles gambiae* Giles and *Anopheles stephensi* Liston), with LC_50_ values ranging from 4.42 to 16.80 μg/mL. The bio-guided EO fractionation identified furanosesquiterpenes as the main larvicidal compounds. A GC–MS-driven untargeted metabolomic analysis revealed 32 affected metabolic pathways in treated larvae. The EO non-target toxicity on *Daphnia magna* Straus (LC_50_ = 4.51 μL/L) and its cytotoxicity on a human kidney cell line (HEK293) (IC_50_ of 14.38 μg/mL) were also assessed. This study shows the potential of plant products as innovative insecticidal agents and lays the groundwork for the possible exploitation of *C. myrrha* EO in sustainable approaches for mosquito management.

**Graphical Abstract:**

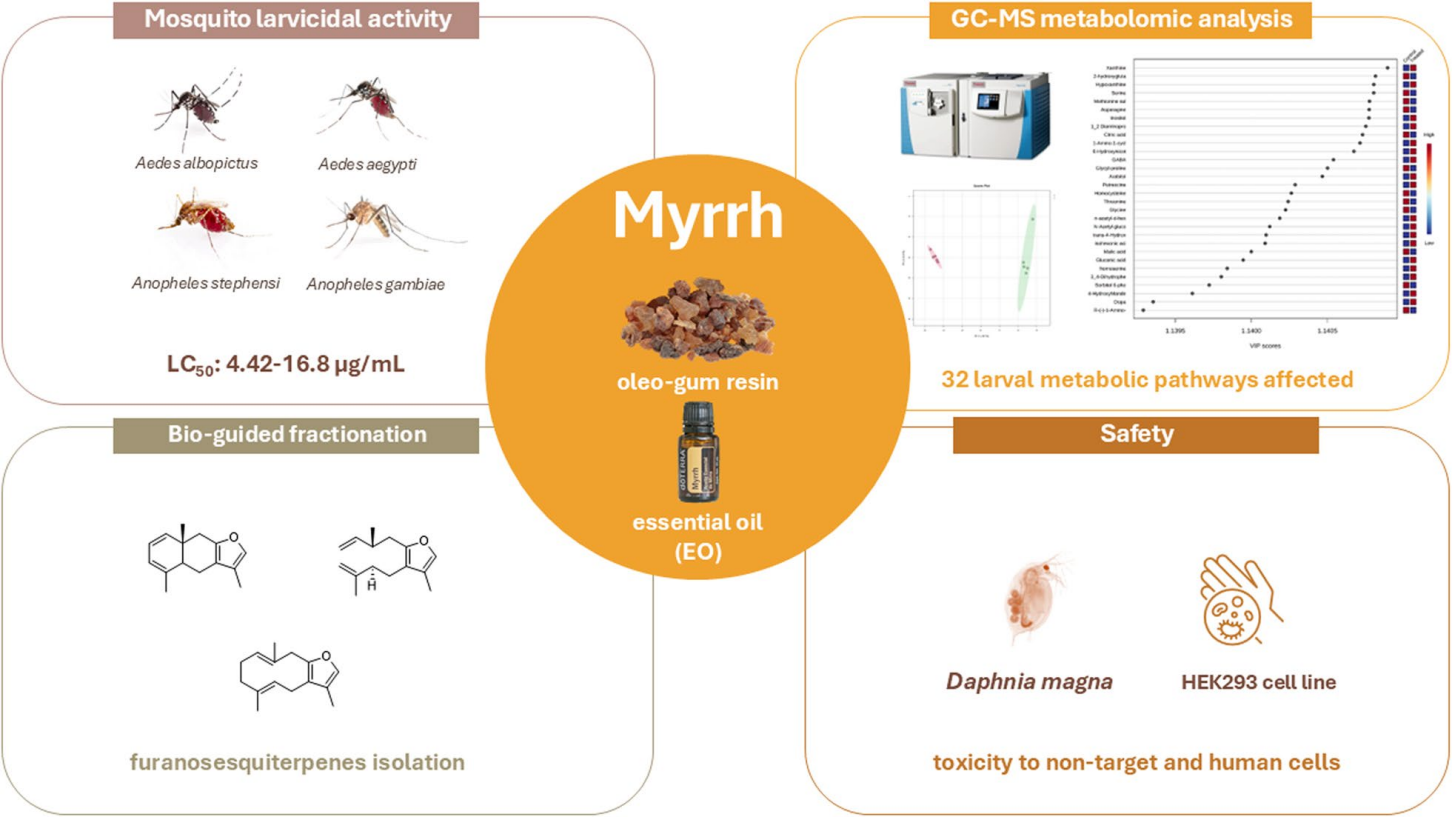

**Supplementary Information:**

The online version contains supplementary material available at 10.1007/s13659-024-00492-6.

## Introduction

The rapid and continuous emergence of vector-borne diseases (VBDs) is at the heart of public health concern worldwide [1–3]. Although conventional insecticides have been largely employed, their overuse has led to negative effects on human and environmental health and has contributed to the rise of insecticide resistance [4, 5]. From this derives the urgent need to discover effective, novel, safe, and eco-friendly products, and essential oils (EOs) are raising considerable interest as sources of bioactive compounds employable as insecticides [6]. *Commiphora myrrha* (T.Nees) Engl. (syn. *Commiphora molmol* (Engl.) Engl. ex Tschirch), commonly known as myrrh, is a sturdy, gray-barked shrub belonging to the Burseraceae family, largely distributed in South-Arabia and North-East Africa. Its long history of use as a medicinal plant is attributed to its oleo-gum resin [7], consisting of a water-soluble gum, alcohol-soluble resin, and a fluid fraction made up of volatile compounds [8], that are mainly represented by furanosesquiterpenes [9]. Nowadays, the oleo-gum resin of myrrh finds many applications in pharmaceuticals, cosmetics, foods, and herbal products [10], and this is also linked to its relatively low price on the market (11.00–15.00 $/kg for myrrh obtained in Africa) [11]. The oleo-gum resin is traditionally employed to treat several health issues [8] and has been studied for its significant antiseptic, anesthetic, and antitumor properties [12]. Additionally, myrrh is employed as flavoring in alcoholic and non-alcoholic beverages, with a maximum permitted level of 0.025%, as well as in dairy products, frozen, sweet, baked goods, and meat and derivatives [10].

The insecticidal and acaricidal activity of *C. myrrha* EO has scarcely been investigated, with studies limited to its effects on the food-stored product beetle *Sitophilus oryzae* (L.) and the two-spotted spider mite *Tetranychus urticae* Koch [13, 14]. However, no data is currently available regarding the efficacy of the EO on mosquito vectors. This study aimed to evaluate the larvicidal activity of myrrh EO on Culicidae of public health relevance as *Aedes albopictus* Skuse, *Ae. aegypti* L., *Anopheles gambiae* Giles, and *An. stephensi* Liston. Additionally, a bio-guided fractionation of the EO was performed to verify the involvement of furanosesquiterpenes in its insecticidal action. An untargeted metabolomic analysis was conducted to assess the possible metabolic pathways affected by the treatment. Furthermore, non-target toxicity was assessed on *Daphnia magna* Straus, and cytotoxicity was examined on human non-tumoral embryonic kidney 293 cells (HEK293).

## Results and discussion

### GC–MS analysis of the essential oil and its fractions

The *C. myrrha* EO was chemically characterized through GC–MS analysis, with a total of identified compounds of 96.50% (Table 1). The EO resulted mainly dominated by furanosesquiterpenes (87.37%), followed by sesquiterpene hydrocarbons (8.04%), oxygenated sesquiterpenes (1.06%), and other compounds in minor amounts. Furanoeudesma-1,3-diene was the most abundant furanosesquiterpene (41.40%), followed by curzerene (25.89%), lindestrene (13.09%), and isofuranodiene (2.00%). Among the sesquiterpene hydrocarbons, *β*-elemene (2.87%) and germacrene B (2.11%) were the major components. The chemical composition of this EO is linear with those reported for EO from Ethiopian plants [15, 16]. Furanoeudesma-1,3-diene was consistently the major compound across all samples, with percentages ranging from 34 to 39% of the total composition, followed by isofuranodiene (20%) [15], and lindestrene (12–14%) [15, 16]. In contrast, the chemical composition differs from that reported by Morteza-Semnani et al. [17], that found curzerene (40%) as the dominant compound. However, the high levels of curzerene in the EO are misleading since, during GC–MS analysis, isofuranodiene is converted into curzerene through the Cope rearrangement [18]. For this reason, the GC–MS quali-/quantitative analysis of isofuranodiene is generally inappropriate.

**Table 1:** Chemical composition of the essential oil (EO) of *Commiphora myrrha*

No	Compound^a^	Chemical class^b^	RI^c^	RI Lit^d^	Area% ± SD^e^	Id^f^
1	(*E*)-*β*-ocimene	MH	1046	1044	0.02 ± 0.01	RI, MS
2	*δ-*elemene	SH	1337	1335	0.35 ± 0.02	RI, MS
3	*α-*copaene	SH	1374	1374	0.02 ± 0.01	RI, MS
4	*β-*bourbonene	SH	1383	1387	0.14 ± 0.01	RI, MS
5	*β-*elemene	SH	1391	1389	2.87 ± 0.03	RI, MS
6	(*E*)-caryophyllene	SH	1418	1417	0.28 ± 0.01	RI, MS
7	*γ-*elemene	SH	1433	1434	0.39 ± 0.02	RI, MS
8	*α-*humulene	SH	1452	1452	0.06 ± 0.01	RI, MS
9	*γ-*muurolene	SH	1475	1478	0.08 ± 0.02	RI, MS
10	germacrene D	SH	1480	1484	0.53 ± 0.03	RI, MS
11	*β-*selinene	SH	1485	1489	0.41 ± 0.02	RI, MS
12	*α-*selinene	SH	1494	1498	0.34 ± 0.00	RI, MS
13	curzerene	FS	1496	1499	25.89 ± 0.46	Std, RI, MS
14	*α-*muurolene	SH	1499	1500	0.07 ± 0.00	RI, MS
15	(*Z*)-*α*-bisabolene	SH	1502	1506	0.03 ± 0.00	RI, MS
16	*γ-*cadinene	SH	1513	1513	0.15 ± 0.02	RI, MS
17	*δ-*cadinene	SH	1523	1522	0.17 ± 0.02	RI, MS
18	selina-3,7-(11)-diene	SH	1540	1545	0.04 ± 0.01	RI, MS
19	germacrene B	SH	1556	1549	2.11 ± 0.08	RI, MS
21	curzerenone	FS	1604	1605	0.07 ± 0.02	RI, MS
22	furanoeudesma-1,3-diene	FS	1625	–	41.40 ± 0.39	Std, MS
23	lindestrene	FS	1632	1623^ g^	13.09 ± 0.08	RI, MS
24	*epi-α-*cadinol	SO	1640	1638	0.53 ± 0.05	RI, MS
25	unknown furanosesquiterpene^i^	FS	1655	–	0.33 ± 0.08	–
26	atractylon	FS	1660	1657	0.66 ± 0.07	RI, MS
27	isofuranodiene	FS	1690	1688^ h^	2.00 ± 0.16	Std, RI, MS
28	germacrone	SO	1694	1693	0.53 ± 0.03	RI, MS
29	(*R*)(*5E*,*9E*)-8-methoxy-3,6,10-trimethyl-4,7,8,11-tetrahydrocyclodeca[b]furan	FS	1719	–	3.93 ± 0.02	MS^i^
	Total identified				96.49 ± 0.27	
	Monoterpene hydrocarbons (MH)				0.02	
	Sesquiterpene hydrocarbons (SH)				8.04	
	Furanosesquiterpenes (FS)				87.37	
	Oxygenated sesquiterpenes (SO)				1.06	

^a^Compounds are listed according to their order of elution from the HP-5MS column; ^b^Chemical class: MH, monoterpenes hydrocarbons; SH, sesquiterpenes hydrocarbons; SO, oxygenated sesquiterpenes; FS, furanosesquiterpenes; ^c^Linear retention index calculated with the Van den Dool and Kratz formula [19]; ^d^Retention index from Adams (2007) [20]; ^e^Relative percentage values derived from two independent analyses. SD, standard deviation; ^f^Method of identification: Std, comparison with available analytical standards; RI, linear with those calculated with ADAMS (2007) [20] and NIST (2020) [21] libraries; MS, correspondence of the mass spectrum with respect to that of ADAMS (2007) [20], FFNSC (2012) [22], and NIST20 [21] libraries; ^g,h^RI comparable with literature [23, 24]; ^i^MS (EI): m/z = 216 (M^+^), 145, 121, 108, 91, 79, 44

Then, the EO was fractionated into two main fractions, which were subsequently analyzed by GC–MS. Fraction 1, with 94.37% of identified compounds, was primarily characterized by sesquiterpene hydrocarbons, with *β*-elemene and germacrene B as the main components (29.08 and 15.81%, respectively). *δ*-Elemene (6.94%), *β*-selinene (6.33%), *α*-selinene (6.12%), *γ*-elemene (5.64%), and germacrene D (4.79%) were also found in minor percentages (Table S2, Supplementary File 1). The GC–MS analysis of fraction 2 confirmed the presence of furanosesquiterpenes contained in the EO, with a total of identified compounds of 99.70%. This fraction was predominantly characterized by furanoeudesma-1,3-diene (44.95%), curzerene (33.90%), and lindestrene (16.67%) (Table S3, Supplementary File 1), with smaller amounts of atractylon and isofuranodiene (1.85 and 1.60%, respectively).

### Purification of furanosesquiterpenes

Fraction 2 (348 mg) was further purified to give 37.7 mg of furanoeudesma-1,3-diene, 13 mg of curzerene, and 8 mg of isofuranodiene. The structures of the furanosesquiterpenes were confirmed by ^1^H, ^13^C NMR, MS spectrometry, and IR spectroscopy aligning with published data [25–27]. The full characterization of the isolated compounds is reported in Supplementary File 1 along with the NMR spectra for furanoeudesma-1,3-diene (Supplementary File 1, Fig. S2, S3, S4).

### HPLC–DAD quantitative analysis

**Fig. 1:**
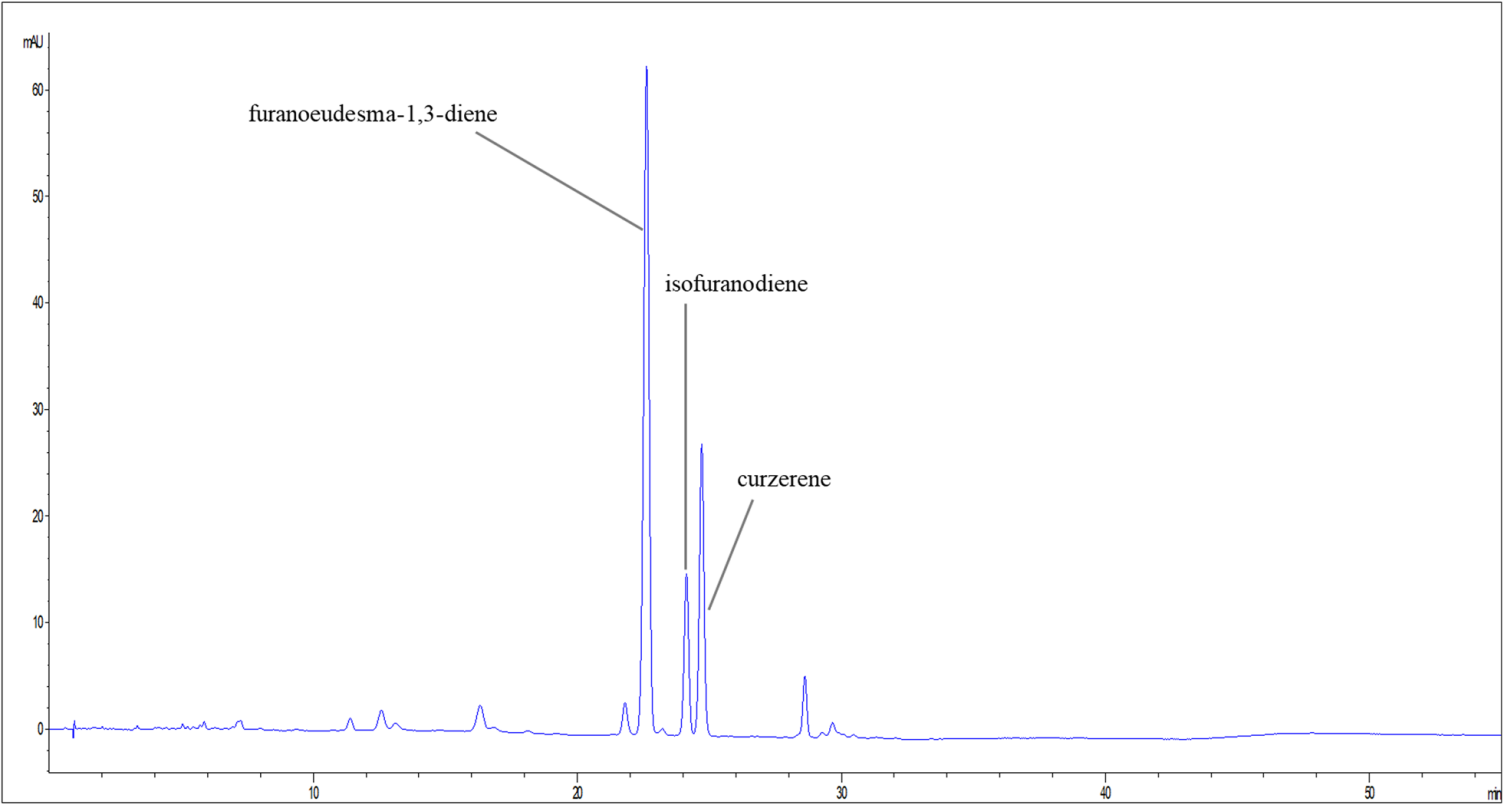
HPLC–DAD chromatogram of *Commiphora myrrha* essential oil (EO)

The main furanosesquiterpenes of *C. myrrha* EO and fraction 2, namely furanoeudesma-1,3-diene, isofuranodiene, and curzerene, were quantified through HPLC–DAD analysis (Fig. 1) to avoid the thermal degradation of isofuranodiene and to have a reliable quantification of the compounds. In detail, furanoeudesma-1,3-diene was confirmed as the predominant furanosesquiterpene (68.42 g/100 g EO), followed by curzerene (18.03 g/100 g EO), and isofuranodiene (7.40 g/100 g EO). Similar levels were also found in fraction 2, as reported in Table S4, Supplementary File 1.

These results are consistent with those obtained from the GC–MS quali-/quantitative analysis, where furanoeudesma-1,3-diene was identified as the principal furanosesquiterpene. The presence of curzerene, also confirmed by HPLC–DAD analysis, may be attributed to the extraction protocol applied to *C. myrrha* oleo-gum resin to obtain the EO, which could have led to the formation of high amounts of curzerene through thermal degradation of isofuranodiene. Further details on the quantification of the furanosesquiterpenes are provided in Supplementary File 1 (Table S4).

### Mosquito larvicidal activity

This study aimed to assess the larvicidal potential of *C. myrrha* EO on four mosquito species of significant health impact and to identify the class of compounds primarily responsible for the activity. Both *C. myrrha* EO and its furanosesquiterpene fraction proved to be highly effective against all the mosquito species tested (LC_50_ from 4.42 to 16.81 and from 3.72 to 5.04 μg/mL, respectively) (Table 2).

**Table 2:** Larvicidal activity of the *Commiphora mhyrra* essential oil (EO), and its derived products against *Anopheles* and *Aedes* mosquito species

Mosquito species	Tested product	LC^a^	Concentration (μg/mL) (95% CI^b^)	Slope (± SE^c^)	Intercept (± SE)	*χ*^2^, *df*, *p*-value
*Aedes albopictus*	EO^d^	10	8.71 (7.34–9.80)	4.49 ± 0.47	− 5.50 ± 0.57	22.04, 18, 0.230 ns
		30	12.85 (11.77–13.75)			
		50	16.81 (15.83–17.93)			
		90	32.42 (28.42–39.33)			
	Terpenes fraction	–	No mortality at the maximum tested concentration (100 μg/mL)	–	–	–
	Furanosesquiterpenes fraction	10	2.24 (1.83–2.59)	3.64 ± 0.30	− 2.55 ± 0.23	16.02, 18, 0.591
		30	3.61 (3.20–3.98)			
		50	5.04 (4.63–5.45)			
		90	11.34 (10.00–13.37)			
*Anopheles gambiae*	EO	10	4.57 (2.80–6.01)	3.42 ± 0.50	− 3.54 ± 0.59	7.13, 18, 0.989
		30	7.61 (5.71–8.98)			
		50	10.82 (9.24–11.97)			
		90	25.63 (22.24–32.64)			
	Terpenes fraction	–	No mortality at the maximum tested concentration (100 μg/mL)	–	–	–
	Furanosesquiterpenes fraction	10	1.10 (0.74–1.43)	2.55 ± 0.26	− 1.38 ± 0.19	19.25,18, 0.376
		30	2.17 (1.72–2.57)			
		50	3.49 (3.01–3.92)			
		90	11.07 (9.35–14.01)			
*Anopheles stephensi*	EO	10	5.28 (3.44–6.72)	3.40 ± 0.49	3.74 ± 0.58	23.82, 18, 0.161
		30	8.81 (6.99–10.12)			
		50	12.57 (11.21–13.66)			
		90	29.96 (25.37–38.88)			
	Terpenes fraction	–	No mortality at the maximum tested concentration (100 μg/mL)	–	–	–
	Furanosesquiterpenes fraction	10	1.43 (1.06–1.76)	2.93 ± 0.27	− 1.73 ± 0.20	15.79, 18, 0.607
		30	2.59 (2.16–2.96)			
		50	3.91 (3.48–4.32)			
		90	10.71 (9.25–13.05)			
*Aedes aegypti*	EO	10	1.48 (0.83–2.08)	2.70 ± 0.37	− 1.74 ± 0.32	5.29, 18, 0.998
		30	2.83 (1.99–3.49)			
		50	4.42 (3.60–5.06)			
		90	13.17 (11.19–17.10)			
	Terpenes fraction	–	No mortality at the maximum tested concentration (100 μg/mL)	–	–	–
	Furanosesquiterpenes fraction	10	1.56 (0.63–2.27)	3.41 ± 0.74	− 1.94 ± 0.54	14.15, 14, 0.439
		30	2.61 (1.53–3.26)			
		50	3.72 (2.80–4.23)			
		90	8.83 (7.43–13.24)			
	Furanoeudesma-1,3-diene	10	2.066 (1.55–2.41)	6.40 ± 0.80	− 3.30 ± 0.47	25.26, 18, 0.118
		30	2.71 (2.29–2.98)			
		50	3.28 (2.98–3.49)			
		90	5.20 (4.76–6.06)			
	Isofuranodiene	10	2.74 (2.33–3.08)	4.15 ± 0.36	− 3.09 ± 0.27	13.68, 22, 0.912
		30	4.17 (3.82–4.47)			
		50	5.58 (5.25–5.93)			
		90	11.36 (10.09–13.36)			
	Curzerene	10	2.72 (2.12–3.19)	2.93 ± 0.35	− 2.56 ± 0.27	7.97, 22, 0.997
		30	4.93 (4.44–5.37)			
		50	7.44 (6.78–8.40)			
		90	20.33 (15.83–30.19)			

^a^LC, lethal concentrations that kill 10%, 30%, 50% and 90% of exposed larvae, respectively; ^b^95% CI, lower and upper limits of the 95% confidence interval; ^c^SE, standard error; ^d^EO, essential oil

While mortality rates increased with higher concentrations of both the furanosesquiterpene fraction and EO, the former proved to be more effective against all the tested species. However, the furanosesquiterpene fraction achieved a higher mortality rate at lower doses if compared with the EO (Fig. S5, Supplementary File 1), and LC_10_, LC_30_, LC_50_, and LC_90_ values were reached with lower doses (Fig. S6, Supplementary File 1).

**Fig. 2:**
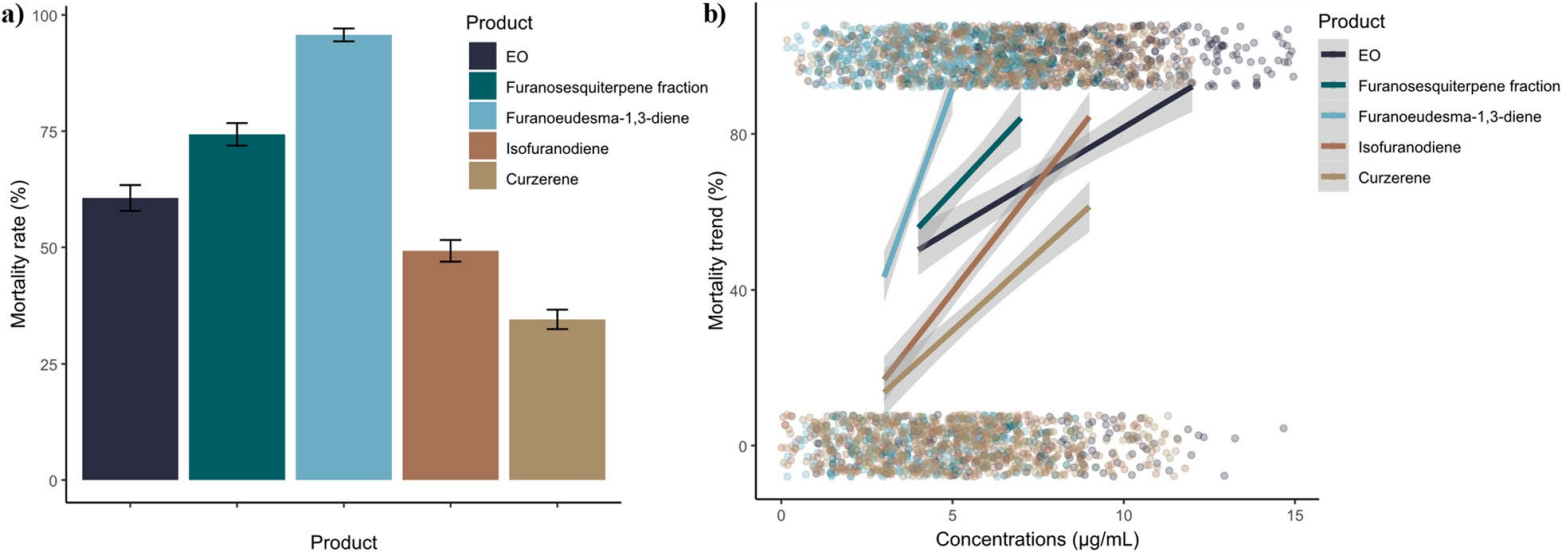
**a** Overall species mortality rate of *Aedes aegypti* treated with the essential oil (EO), the furanosesquiterpene fraction, furanoeudesma-1,3-diene, isofuranodiene, and curzerene, regardless of the tested concentrations. **b** Mortality trend of *Aedes aegypti* by increasing the concentration of the essential oil (EO), the furanosesquiterpene fraction, furanoeudesma-1,3-diene, isofuranodiene, and curzerene. Colored dots indicate the total number of tested individuals clustered around 0 when alive or around 1 when dead.

Regarding pure compounds, they were tested on *Ae. aegypti* since this species showed the highest sensitivity to the EO treatment among the four target vectors. Furanoeudesma-1,3-diene resulted to be the most effective (LC_50_ 3.28 µg/mL), followed by isofuranodiene (LC_50_ 5.58 µg/mL), and curzerene (LC_50_ 7.44 µg/mL) (Table 2, Fig. 2a) *(GLMM post-hoc with Bonferroni correction—Furanoeudesma-1,3-diene vs. Isofuranodiene: OR* = *22.983, SE* = *8.058, z* = *8.941, p* < *0.0001; Furanoeudesma-1,3-diene vs. EO: OR* = *14.484, SE* = *5.147, z* = *7.478, p* < *0.0001; Furanosesquiterpenes fraction vs. Furanoeudesma-1,3-diene: OR* = *0.129, SE* = *0.046, z* = *-5.655, p* < *0.0001; Curzerene vs. Furanoeudesma-1,3-diene: OR* = *0.023, SE* = *0.008, z* = *-10.679, p* < *0.0001)*. Concerning the mortality rates of all the tested products, furanoeudesma-1,3-diene reached the highest mortality rate with the lowest doses if compared with the others, and even a slight increase in its concentration led to a significantly faster mortality trend (Fig. 2b).

This is the first study that reports the larvicidal activity of *C. myrrha* EO and demonstrates the key role of furanosesquiterpenes in its efficacy. Furthermore, all the tested products exhibited strong activity against the treated mosquito larvae, with LC_50_ values below 20 μg/mL.

The extracts derived from species of the genus *Commiphora* Jacq. have been previously tested on mosquitoes. Indeed, the extract deriving from *C. myrrha* resin showed an LC_50_ of 281.83 μg/mL on *Ae. aegypti* larvae [28], while *C. caudata* (Wight & Arn.) Engl. extracts exhibited mild toxicity on *Ae. aegypti*, *An. stephensi*, and *Culex quinquefasciatus* Say (LC_50_ ranging from 94.76 to 112.85 μg/mL) [29]. Comparable results were also obtained for the EO from *C. berryi* (Arn.) Engl. (LC_50_ ranging from 122 to 175 μg/mL) [30]. Conversely, extracts from *C. swynnertonii* Burtt resin displayed lower LC_50_ values (ranging from 3.95 to 27.04 μg/mL) on *An. gambiae*, *Cx. quinquefasciatus*, and *Ae. aegypti* [31]. The EO from *C. erythraea* (Ehrenb.) Engl. showed a higher toxicity against *Cx. pipiens* L., *Cx. restuans* Theobald, and *Ae. aegypti*, with LC_50_ values ranging from 10.05 to 29.83 μg/mL [32]. It is worthy of notice that the above-mentioned extracts from *Commiphora* species are chemically different from the EO tested in this work, being the latter more concentrated in bioactive furanosesquiterpenes.

Regarding other botanical products, Pavela [33] demonstrated that, although many studies report the mosquito larvicidal activity of EOs, only some of them (i.e., *Cinnamomum microphyllum* Ridl., *C. mollissimum* Hook. F., *C. rhyncophyllum* (Miq.), *Callitris glaucophylla* Joy Thomps. & L.P. Johnson, *Auxemma glazioviana* Taub., *Blumea densiflora* D.C., and *Zanthoxylum oxyphyllum* Edgew.) displayed LC_50_ below 10 μg/mL [33]. Indeed, among them, only the EOs from *A. glazioviana* (LC_50_ of 3 μg/mL against *Ae. aegypti*) [34] and *C. glaucophylla* (LC_50_ = 0.7 μg/mL against *Ae. aegypti*) [35] showed an efficacy like that reported in this work. Overall, our results demonstrate that *C. myrrha* EO is an effective product against mosquito larvae.

### GC–MS-driven untargeted metabolomic analysis

**Fig. 3:**
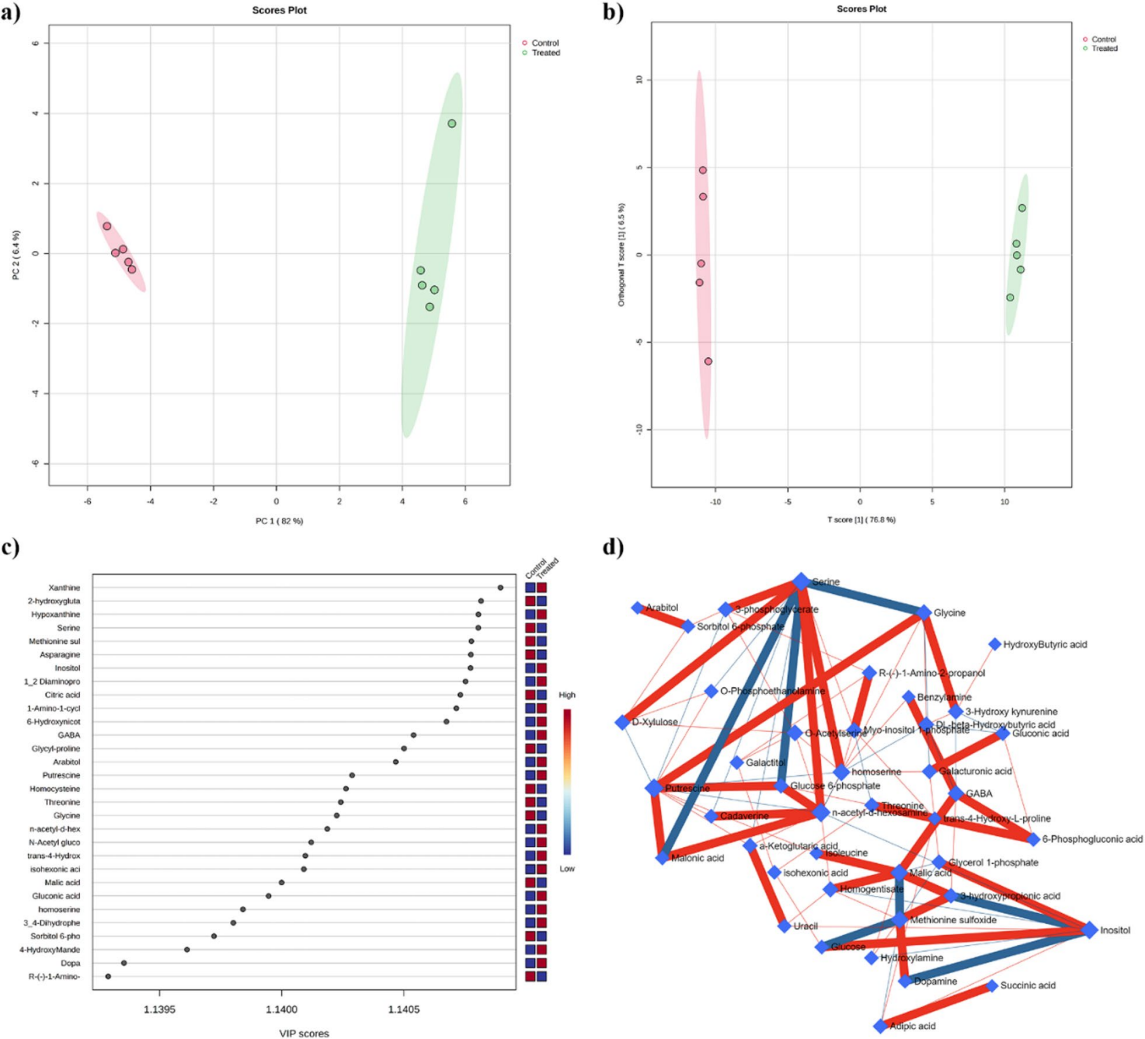
**a** PCA scores plot between the selected PCs. The explained variances are shown in brackets; **b** OPLS-DA score plot of all metabolite features; **c** VIP scores derived from the OPLS-DA model; **d** Correlation network obtained by DSPC algorithm using metabolites the metabolites discriminating treated from untreated larvae, blue nodes represent metabolites, red lines indicate a positive correlation between metabolites. In contrast, the blue lines indicate a negative correlation. N = 5

The untargeted metabolomic analysis allowed the extraction of 565 ions, 172 of which were putatively annotated and belonged to different classes of compounds such as amino acids, organic acids, sugars and sugar alcohols, and fatty acids among others (Supplementary File 2, Table S7). Data were first explored by unsupervised PCA (Fig. 3a) built under the first two PCs, which explained 90.4% of the total variance (82% of the PC1 and 8.4% of the PC2). As reported by the PCA loading plots, the PC1 was mainly dominated by compounds such as spermidine, 6-hydroxy nicotinic acid, xanthine, hypoxanthine and asparagine, whereas the PC2 by 3-hydroxy propionic acid, 4-hydroxybutyric acid, glycolic acid, 3-phosphoglycerate and lactic acid (Supplementary File 2, Table S7).

The good separation highlighted by PCA allowed further data processing through supervised discriminant multivariate analysis such as OPLS-DA, which is specifically designed to identify variables that contribute to the differentiation between these groups, focusing on the variations that are directly related to group discrimination and enhancing the ability of the model to identify and highlight differences between groups [36].

As reported in Fig. 3b, the validated (Supplementary File 2, Table S7) OPLS-DA model confirmed the separation of the control and treated groups, indicating that their differences were statistically reliable (Fig. 3b). Further, the VIP scores analysis allowed us to identify those compounds that could be considered the main drivers of group separation (Fig. 3c). The model identified 106 compounds with a VIP score higher than 1 (Supplementary File 2, Table S7) and the top 30 were reported in Fig. 3c. Among them, metabolites such as xanthine, 2-hydroxyglutaric acid, hypoxanthine, serine, methionine sulfoxide, and asparagine, among others, were characterized by the highest VIP scores (Fig. 3c).

DSPC is a machine-learning algorithm that discovers the connections between many metabolites derived from few samples [37]. Considering that the low number of samples could be a limitation to the significance of our analysis, we used the DSPC to produce an additional validation of our results. Figure 3d shows the graphical representation of the DSPC results applied to the metabolite intensities obtained from control and treated larvae. Nodes correspond to the metabolites, and the edges represent the correlations among them. Red edges indicate positive correlations, and blue edges are negative correlations. Each node representing a metabolite is defined by its degree and betweenness values (Table 3) [37].

**Table 3:** DSPC network analysis.

Label	Degree	Betweenness
*N*-acetyl-D-hexosamine	11	175.48
Putrescine	11	107.35
Serine	11	106.57
Inositol	9	140.43
homoserine	8	90.05
Malic acid	8	60.76
Methionine sulfoxide	8	45.73
GABA	6	68
Glycine	6	65.52
O-Acetylserine	6	57.01
Myo-inositol 1-phosphate	5	53.71
3-Hydroxy kynurenine	5	48.12
3-phosphoglycerate	5	46.93
D-Xylulose	5	39.69

The degree of node and betweenness of the interrelationships were calculated on the annotated metabolites in control and treated larvae

In the table are reported only those metabolites characterized by a Degree threshold > of 5 and a betweenness higher than 35. The full list of metabolites is reported in Supplementary File 2, Table S7. N = 5

The degree indicates the number of connections that a node has with other nodes, while betweenness measures the number of interconnections. Nodes with high degree and betweenness values are likely to be significant hubs [38]. We used a degree threshold greater than 5 and betweenness greater than 35 to identify metabolites characteristic of the lethal phenotype. Network analysis of control and treated larvae reveals that metabolites primarily involved in amino acid, polyamine, organic acid, and sugar metabolism, such as *N*-acetyl-D-hexosamine, putrescine, serine, inositol, homoserine, and malic acid, among others, play a central role in the mode of action of EOs (Fig. 3c and Table 3). Moreover, the results highlighted that: ***i***) *N*-acethyl-D-hexosamine was positively correlated with serine, glucose-6-phosphate, cadaverine and malonic acid; ***ii***) putrescine was positively correlated with glucose-6-phosphate, malonic acid and glycine, whereas ***iii***) serine was negatively correlated with glycine, malonic acid and glucose-6-phosphate, and positively correlated with 3-phosphoglycerate, xylulose, homoserine and *N*-acetyl-hexosamine (Fig. 3d).

**Fig. 4:**
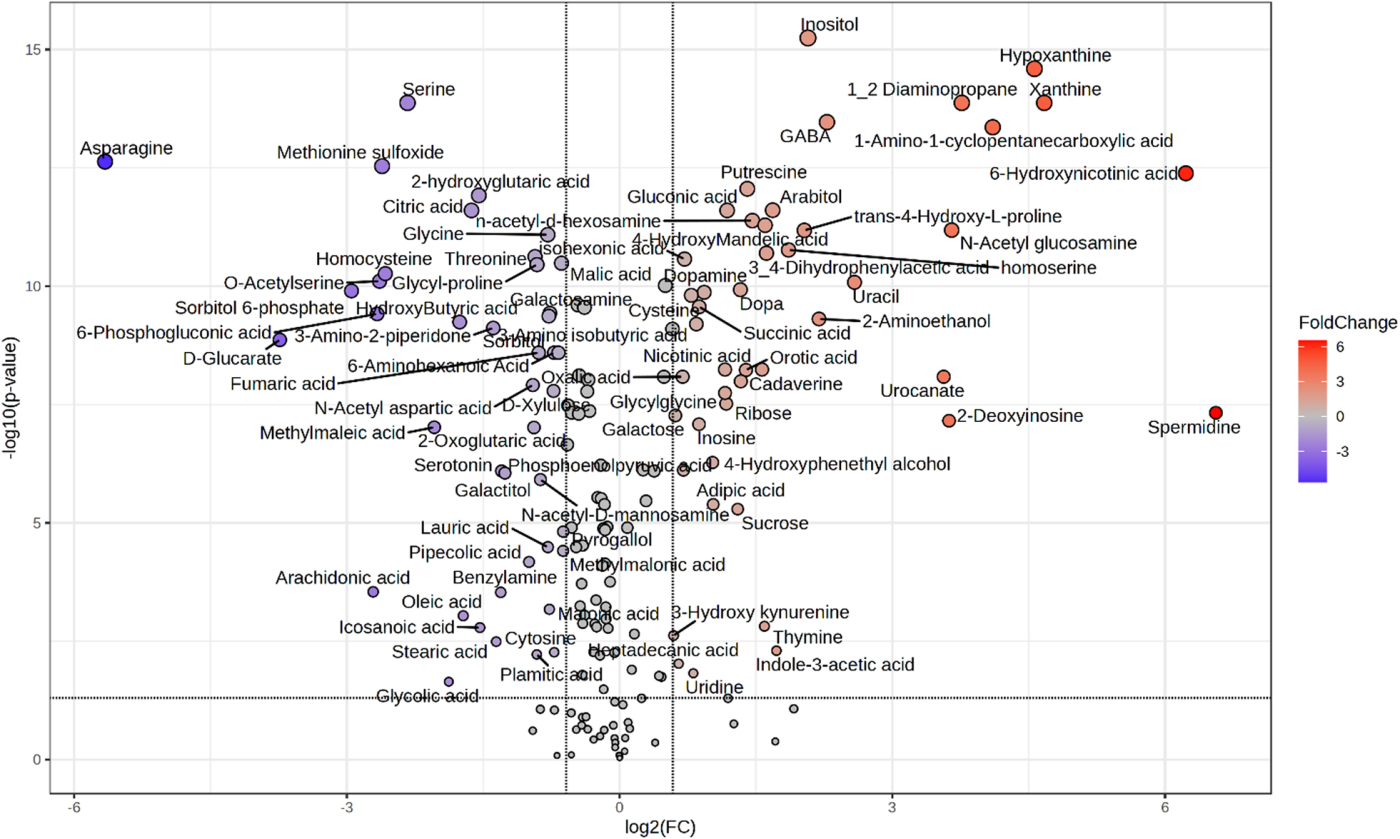
Important features selected by volcano plot with fold change threshold > 1.5 and t-tests threshold ≤ 0.05. The red and violet circles represent features above the threshold. Note both fold changes and *P* values are Log_10_ transformed. The further its position away from the (0), the more significant the feature is N = 5

After multivariate and DSPC analysis the annotated metabolites were further analyzed through univariate analysis to highlight all the differentially accumulated metabolites between control and treated larvae (Supplementary File 1 Table S7 and Fig. 4). The t-test univariate analysis revealed that the treatment significantly impacted 137 out of 172 metabolites. Specifically, 56 metabolites showed significant accumulation in treated larvae, while 81 were significantly reduced (Supplementary File 2, Table S7). Further analysis was conducted using Fold Change (FC) with a cutoff of 1.5 (Supplementary File 2, Table S7) to focus on the most strongly affected metabolites (Supplementary File 2, Table S7). These results were then integrated using a Volcano plot analysis, applying a t-test *p*-value of ≤ 0.05 and an FC > 1.5, as depicted in Fig. 4 and detailed in Supplementary File 1, Table S5. The volcano plot allowed the reduction of the number of affected metabolites to 87, of which 42 were significantly reduced and 45 significantly increased (Fig. 4 and Supplementary File 1, Table S5).

Among the analyzed compounds, there was a significant accumulation of polyamine spermidine, purines xanthine and hypoxanthine, gamma-aminobutyric acid (GABA), etc. In contrast, the levels of certain compounds, such as the amino acids asparagine and serine, and the organic acid glucaric acid, were significantly reduced in response to the treatment (Fig. 4).

**Fig. 5:**
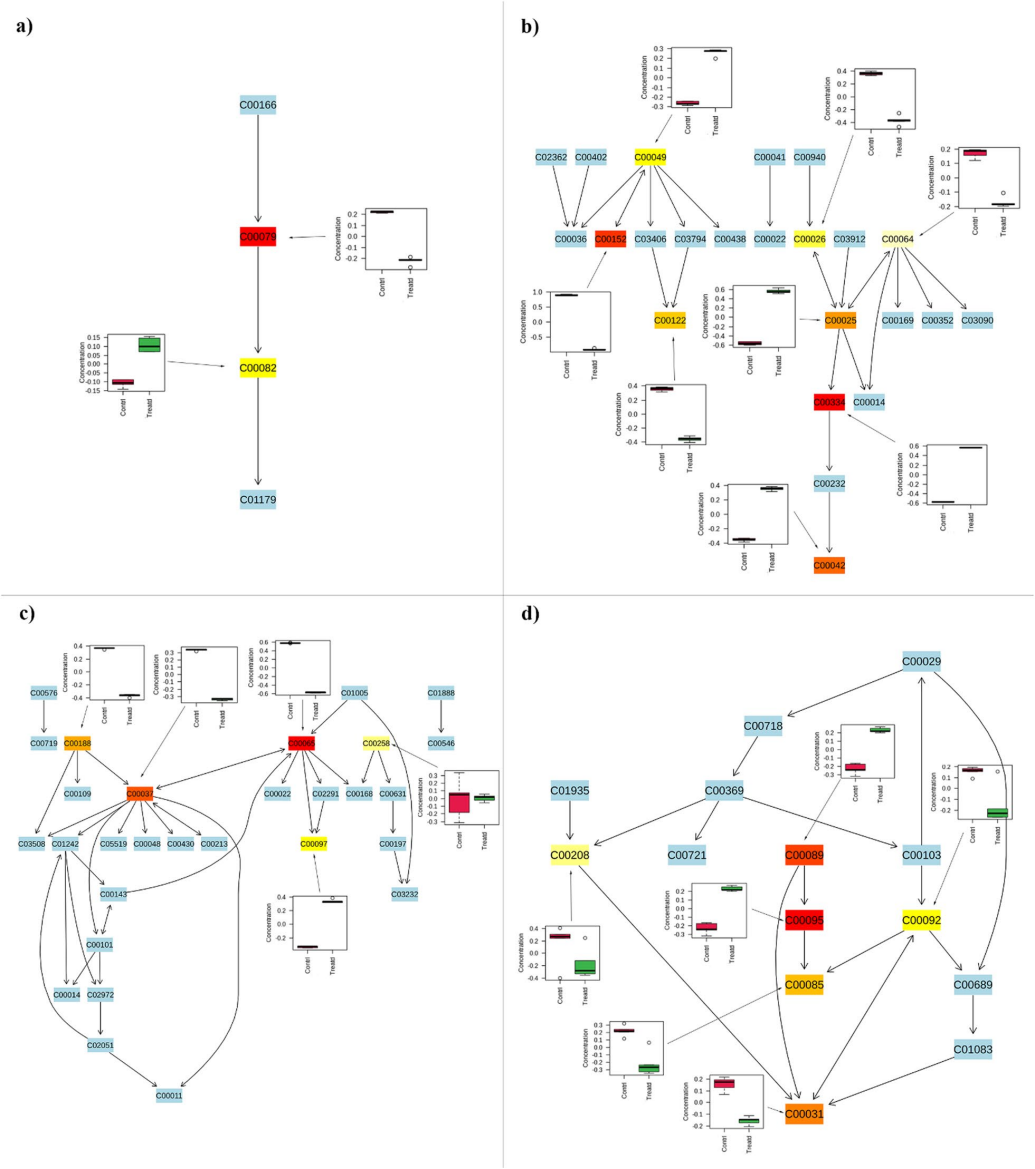
Here are the first four most affected pathways identified in the pathway analysis (Table 3), along with the full list of associated metabolites. Metabolites that belong to these pathways but were not identified during the analysis are highlighted in light blue. The colored boxes, ranging from light yellow to red, represent the statistical significance of the identified metabolites (light yellow indicates a P value closer to 0.05, while red indicates a P value greater than 0.001). Box plots show the trend of each metabolite identified as statistically significant by the t-test, with red boxes representing control larvae and green boxes representing treated larvae. Each code within the boxes corresponds to the KEGG code of the metabolites associated with the pathways; **a** Phenylalanine tyrosine and tryptophan biosynthesis (C00079—L-Phenylalanine, C00082—L-Tyrosine); **b** Alanine aspartate and glutamate metabolism (C00025—Glutamic acid, C00026—alpha-Ketoglutaric acid, C00042—Succinic acid, C00049—Aspartic acid, C00064—L-Glutamine, C00122—Fumaric acid, C00152—L-Asparagine, C00334—GABA), **c** Glycine serine and threonine metabolism (C00037—Glycine, C00065—Serine, C00097—L-Cysteine, C00188—Threonine, C00258—Glycerate), **d** sucrose metabolism (C00031—D-Glucose, C00085—D-Fructose 6-phosphate, C00092—D-Glucose 6-phosphate, C00095—D-Fructose, C00089—Sucrose, C00208—Maltose)

Finally, the data were analyzed through pathway analysis to highlight which pathways were significantly and highly impacted by the EO treatment, considering our metabolomic coverage. The results highlighted that 32 pathways were significantly affected in response to EO treatment (Supplementary File 2, Table S7), but only 21 were characterized by an impact higher than 0.2 (Supplementary File 1, Table S6). Among them, the pathway related to the phenylalanine tyrosine and tryptophan biosynthesis was the most affected highlighting an impact of 1. It was followed by several pathways involved in amino acid biosynthesis (i.e., alanine aspartate and glutamate metabolism, glycine serine and threonine metabolism, arginine and proline metabolism, among others) and sugars metabolism (i.e. sucrose metabolism, fructose and mannose metabolism, glycerolipids metabolism, etc.) (Supplementary File 1, Table S6) (Fig. 5).

This research presents a comprehensive study examining the metabolic impact of *C. myrrha* EO treatment on *Ae. aegypti* larvae through GC–MS-driven untargeted metabolomic analysis. The analysis suggested that the EO triggered a broad spectrum of metabolic responses. In detail, one of the most impacted metabolisms was that of amino acids and significant changes were observed in pathways related to alanine, aspartate, and glutamate, as well as glycine, serine, and threonine metabolism. A similar metabolic response was found in *Tribolium castaneum* (Herbst) larvae and *Drosophila melanogaster* (Meigen) [39] adults after the treatment with organophosphorus pesticides and pyrethroids, respectively. The increase in amino acids suggested protein degradation since, as also reported in our work, an increase in free *N*-acetylamino acids that are only produced post-translationally by protein *N*-acetylation was observed [39]. Moreover, in our study, it was observed a decrease in tryptophan, a pivotal molecule involved in protein biosynthesis and whose down-accumulation has been observed in response to several insecticide treatments [39, 40]. In addition, Brinzer et al. [39] further demonstrated that tryptophan catabolism, one of the most impacted pathways in our experiment, is crucial in the defense of the organism against insecticides [39]. The degradation of the above-mentioned amino acid usually leads to an accumulation of neurotoxic compounds [39, 41], such as 3-hydroxykynurenine, which has also been observed in our study after the EO treatment. The reduction of fumaric acid and phenylalanine observed in this study is consistent with what was previously observed by Gao et al. [40]. Indeed, they detected a reduction of these metabolites in the moth *Spodoptera frugiperda* (JE Smith) exposed to the LD_90_ of the insecticide spinetoram, and they suggested that the downregulation of phenylalanine may reduce fumaric acid levels, slowing the tricarboxylic acid (TCA) cycle and decreasing guanosine triphosphate production under spinetoram-induced stress.

Besides the changes observed in phenylalanine, tyrosine, and tryptophan, which are end products of glycolysis and aromatic amino acids, in treated larvae it was also observed a general reduction in glycine, serine and threonine content whose metabolism is linked to glucose, glycogen, and pyruvate metabolism, all of which are integral to energy metabolism [41–44]. The potential alteration of energetic metabolism was confirmed by the impact on sucrose metabolism, the pentose phosphate pathway, and the TCA cycle. Their downregulation could be confirmed by the reduction in glycolytic phosphorylated sugars (i.e. glucose-6-phosphate, fructose-6-phosphate), an accumulation of those belonging to the pentose phosphate pathway (i.e. ribulose-5-phosphate), and the activation of purine metabolism.

The latter has also been observed in *An. sinensis* Wiedemann larvae in response to the treatment with deltamethrin [42] and has been reported to generate oxygen species (ROS), which could result in DNA damage and apoptosis in the affected individuals [42].

Insecticides, particularly neurotoxic compounds like permethrin, have been shown to disrupt normal physiological processes in *Anopheles* larvae, resulting in oxidative stress characterized by increased ROS levels. This oxidative stress can lead to cellular damage and, ultimately, death of the larvae [63, 64]. The accumulation of ROS is a well-documented response to various environmental stresses, including exposure to toxic compounds, and plays a critical role in the pathophysiology of insecticide-induced mortality [45, 46]. Earlier research demonstrated that the levels of GABA and polyamines can increase in response to oxidative stress also induced by ROS [47–50]. The relationship between insecticides, ROS accumulation, and the protective roles of osmoprotective compounds like GABA and polyamines, has been investigated by examining the transcriptomic responses of *Anopheles* larvae to insecticide exposure. RNA sequencing analyses have revealed significant changes in gene expression related to stress response, detoxification, and metabolic processes, indicating that larvae are actively responding to the oxidative stress induced by insecticides [45, 46]. This adaptive response may involve the upregulation of genes associated with the synthesis of osmoprotective compounds, thereby enhancing the ability of larvae to cope with the detrimental effects of ROS.

### Non-target toxicity

*Daphnia magna* is a microcrustacean employed as a model organism for acute and chronic toxicity evaluation of compounds in aquatic ecosystems. Its large employment is due to its high sensitivity to toxic agents [51, 52]. Table 4 reports the toxicity of *C. myrrha* EO, which resulted toxic to *D. magna* with a LC_50_ of 4.51 µL/L, and a LC_90_ of 13.09 µL/L.

**Table 4:** Effects of *Commiphora myrrha* essential oil (EO) on *Daphnia magna*

Tested product	LC	Concentration (CI_95_) (µL/L)^a^	*χ*^2^, *df*, *p*-value^b^
*C. myrrha* EO	50	4.51 (3.30–5.82)	3.158, 4, 0.531
	90	13.06 (9.47–23.02)	

^a^LC_50_ and LC_90_, lethal concentrations values expressed in µL/L, CI_95_ = 95% confidence intervals, essential oil (EO) activity is considered significantly different when the 95% CI fail to overlap; ^b^χ^2^, Chi-square value, not significant (ns) (*p* > 0.05)

The assessment of non-target toxicity of insecticides is of crucial importance for their real-world application. Herein, although the observed toxicity resulted marked, it is quite lower than that of conventional insecticides. For instance, pyrethroids are encompassed with a high toxicity on many non-target organisms. Indeed, the LC_50_ on some fish species at 48 h ranged around 1.6–5.13 µg/L for deltamethrin [53] and 0.05–245.7 µg/L for permethrin [54]. Regarding *D. magna*, the 48 h effective concentrations (EC_50_) are 0.16 µg/L for deltamethrin [55] and 0.2–0.6 µg/L for permethrin [56]. Furthermore, some natural products commercially employed as insecticides showed toxicity on this non-target species. For instance, the toxic effect of neem oil has been assessed on *D. magna* and the EC_50_ (48 h) value was 0.17 mL/L [57].

The present laboratory evaluation of *C. myrrha* EO toxicity on *D. magna* represents a preliminary step and, since insecticides are commonly employed in formulations, further studies are needed to potentially lower *C. myrrha* EO toxicity, for instance through its encapsulation.

### Cytotoxicity

The cytotoxicity of *C. myrrha* EO was assessed on the HEK293 cell line, as reported in Table 5.

**Table 5:** Cytotoxic activity of *Commiphora myrrha* essential oil (EO) on human embryonic kidney cell line (HEK293)

Tested product	IC_50_ (CI_95_) (μg/mL)^a^
*C. myrrha* EO	14.38 (11.50–17.97)
Cisplatin (positive control)	3.96 (3.76–4.17)

^a^IC_50_, the concentration of compound that affords a 50% reduction in cell growth (after 72 h of incubation), CI, confidence interval

The cytotoxicity of this EO has already been reported on squamous cell carcinoma (A431) (IC_50_ of 6.4 μg/mL) and on melanoma cells (RPMI-7951 and SK-MEL-28) (IC_50_ of 6.3 and 9.5 μg/mL, respectively). This cytotoxicity was also demonstrated on human keratinocytes (MBU-IM), with a major selectivity for tumor cell lines [58]. This cytotoxicity is undoubtedly linked to the presence of furanosesquiterpenes. Indeed, furanoeudesma-1,3-diene resulted to be cytotoxic in epidermoid carcinoma (A431) (IC_50_ of 9.9 μg/mL) and in malignant melanoma (RPMI-7951 e SK-MEL-28) (IC_50_ of 7.1 and 12.0 μg/mL, respectively) cell lines [58].

Regarding the cytotoxicity of isofuranodiene, this has been previously reported on different tumor lines such as cervical cancer (HeLa) (IC_50_ of 0.6 μg/mL), laryngeal cancer (Hep-2) (IC_50_ of 1.7 μg/mL), fibrosarcoma (HT-1080) (IC_50_ of 2.2 μg/mL), as well as on the stomach cancer cell line (SGC-7901) (IC_50_ of 4.8 μg/mL) or lung (A549) (IC_50_ of 9.4 μg/mL) [59]. Curzerene is also known for its cytotoxicity on tumor cell lines as human breast adenocarcinoma cells (MDA-MB 231) (IC_50_ of 13.9 μg/mL), glioblastoma (T98G) (IC_50_ of 60.3 μg/mL), and colon carcinoma (HCT116) (IC_50_ of 33.3 μg/mL) [60–62]. Information on the mode of action is mainly reported for isofuranodiene and primarily concerns cancer cell lines. In fact, the compound appears to exert its cytotoxicity by induction of apoptosis, inhibition of cell proliferation, and induction of necrosis [63]. A similar mechanism of action has also been reported for curzerene [64]. In this context, the cytotoxicity of *C. myrrha* EO could potentially be further reduced by using formulations, as has been demonstrated with other EOs [65].

The observed toxicity of the EO to HEK293 cells raises important considerations regarding its potential impact on human health and environmental safety. HEK293 cells are commonly used as a model for assessing cytotoxicity due to their sensitivity and relevance to human biology [66]. The toxicity may suggest that certain components of the EO could interact with cellular pathways, leading to harmful effects at molecular or organ level. From a human health perspective, the toxicity observed in vitro indicates a need for further studies, such as dose–response assessments, to determine safe exposure levels. EOs often contain bioactive compounds, such as terpenes and phenols, which, while beneficial at low concentrations, may exert cytotoxic or even genotoxic effects at higher doses. Regarding environmental impact, the release of toxic substances into ecosystems could harm aquatic and terrestrial organisms. EOs are increasingly being used in biopesticides and cosmetics, and their environmental fate (e.g., biodegradability, bioaccumulation) should be evaluated. Components toxic to human cells might similarly affect non-target organisms, disrupting ecological balance.

## Conclusions

In the complex scenario of mosquito management, the search for innovative products is crucial, and botanicals show a promising potential. Concerning *C. myrrha*, this study highlights the larvicidal potential of its EO on four mosquito species of public health relevance. Moreover, its fractionation allowed the identification of furanosesquiterpenes as the main responsible for the bioactivity. The low LC_50_ values obtained (ranging from 4.42 to 16.80 μg/mL) could pave the way for the potential exploitation of myrrh as an effective larvicidal agent and this could also be favored by the established large-scale production of oleo-gum resin for food, pharmaceuticals, cosmetics, and perfumery applications and by its relatively low price on the market. Further studies are needed to develop formulations based on this EO and to reduce its toxicity on non-target organisms as well as on human cells.

## Materials and methods

### Chemicals and reagents

The mix of C_7_–C_40_ and C_10_–C_40_ alkanes, the solvents employed for GC–MS and HPLC–DAD analysis, as well as those used for the fractionation of the EO were purchased from Merck (Milan, Italy). Deionized water (> 18 MΩ cm resistivity) was purified using a Milli-Q SP Reagent Water System (Millipore, Bedford, MA, USA). The EO was acquired from dōTERRA (Utah, Stati Uniti, L.60205991 v3, https://www.doterra.com). Thin layer chromatography (TLC) was performed using silica gel 60 F254 plates, which were also oxidized with cerium molybdate stain. Column chromatography was conducted using silica gel 60 (70–230 mesh and 200–400 mesh, Merck, Milan, Italy).

### Chemicals instrumentation

^1^H- (500 MHz) and ^13^C-NMR (125 MHz) spectra were acquired with a Bruker Avance III 500 MHz (Billerika, MA). The chemical shifts for ^1^H- and ^13^C-NMR are expressed as *δ* [parts per million (ppm)] and coupling constants are given in hertz. Deuterated chloroform was purchased from Merck (Milan, Italy). The following abbreviations are employed to show the coupling in the spectra reported in the Supplementary File 1: s (singlet), d (doublet), dd (doublet of doublets), ddd (doublet of doublets of doublets), t (triplet), dt (doublet of triplets), q (quartet), m (multiplet). The MS spectra were acquired through an Agilent 8890 GC–MS, coupled to a single quadrupole mass spectrometer (5977B) purchased from Agilent (Santa Clara, California, USA) and equipped with an autosampler PAL RTC120 (CTC Analytics AG, Zwingen, Switzerland). The IR spectra (cm^−1^) were obtained using a Perkin-Elmer FT-IR Spectrum Two UATR spectrometer (Perkin Elmer, Inc., Waltham, MA, USA). Optical rotation power was recorded on a Yasco 2000 digital polarimeter operating at 20 °C.

### GC–MS qualitative analysis of essential oil (EO)

The EO and its fractions were analyzed through the GC–MS reported in the previous section (Chemicals Instrumentation). The EO and its fractions were solubilized in diethyl ether (1:100). The separation, identification, and semi-quantification of their compounds were performed by using the same analytical conditions reported by Gugliuzzo et al. [67].

### HPLC–DAD quantitative analysis

The EO quantitative analysis was performed employing an Agilent Technologies (Palo Alto, CA, USA) HP-1100 series, made of an autosampler and a binary solvent pump, with a DAD detector. The separation was achieved on a Kinetex© PFP 100A column (100 × 4.6 mm i.d., 2.6 μm) from Phenomenex (Torrance, CA). The analytical conditions followed those previously reported [66]. The calibration curves were constructed by injecting different dilutions prepared from the stock solutions of isofuranodiene, curzerene, and furanoeudesma-1,3-diene diluted in acetonitrile, as reported in Supplementary File 1. The analytical method was validated as reported in Supplementary File 1.

### Essential oil (EO) fractionation and purification of furanosesquiterpenes

The EO was fractioned using gravity column chromatography on silica gel (100 g) using *n*-hexane as the mobile phase. The EO (2 g) was loaded into the column by dry charge on silica (1:2 w/w) after its solubilization in dichloromethane. This fractionation process led to the isolation of two main fractions: fraction 1 (0.2 g) and fraction 2 (1.1 g). The main furanosesquiterpenes were purified following a method previously published [68, 69] with minor modifications (Supplementary File 1).

### Mosquitoes

All mosquito colonies were maintained in the insectary of the University of Camerino at 80 ± 5% relative humidity, and at 28 ± 2°C and photoperiod of 12:12 h light:dark (L:D). Adults of *Ae. aegypti* and *Ae. albopictus* were preserved with a 10% sucrose solution, while those of *An. stephensi* and *An. gambiae* with a 5% solution. Larvae were fed following protocols previously reported [70] in deionized water with 0.5% p/v of artificial sea salt.

### Mosquito larvicidal assays

Larvicidal assays were performed following the World Health Organization (WHO) procedures [71], applying some modifications [72]. The EO and its two fractions were diluted in DMSO at a 1:10 ratio, while the pure compounds at a 1:20 ratio to allow their complete solubilization. The tested concentrations were 10–25 μg/mL for the EO, up to 100 μg/mL for fraction 1, 2–10 μg/mL for fraction 2, 2–6 μg/mL for furanoeudesma-1,3-diene, and 2–9 μg/mL for isofuranodiene and curzerene.

### MTT cytotoxicity assay

The antiproliferative potential of the EO on the human embryonic kidney 293 cell line (HEK293) was evaluated through the MTT assay [3-(4,5-dimethyl-2-thiazolyl)-2,5-diphenyl-2H-tetrazoliumbromide] as reported previously by Page et al. [73]. The cells were exposed to different concentrations of the EO (0.78–100 μg/mL) solubilized in EtOH. The anticancer drug cisplatin (Merck, Milan, Italy) (0.01–50 μg/mL) was used as the positive control. The experiments were performed in triplicate. Cytotoxicity is expressed as the concentration of EO inhibiting cell growth by 50% (IC_50_). The detailed procedures are reported in Supplementary File 1.

### GC–MS-driven untargeted metabolomic analysis

Sample extraction and derivatization followed the methodology outlined by Misra et al. [74], using 50 mg of larvae ground in liquid nitrogen and spiked with ribitol (0.2 mg/mL) as an internal standard. The derivatized samples were then analyzed using a Gas Cromatographer (8890 GC System, Agilent, Agilent, Santa Clara, California, USA) coupled to a single quad Mass spectrometer (5977C GC/MSD, Agilent, Santa Clara, California, USA), using a CTC PAL autosampler (CTC Analytics AG Industriestrasse 20 CH-4222 Zwingen Switzerland). The GC was equipped with a 5MS column (30 m × 0.25 mm × 0.25 µm) and a 10 m precolumn. The instrument's temperatures, analytical settings, and the MS-DIAL analysis for baseline filtering, alignment, deconvolution, peak extraction, and annotation adhered to the protocols set by Misra et al. [75]. Quality control and monitoring of instrumental performance and retention index (RI) shifts were ensured by injecting blank solvents, qualitative controls (QC), and *n*-alkane standards (C_10_-C_40_, even-numbered) at regular intervals. Peak annotations were based on retention index (RI) and spectral similarity, using an in-house EI spectral library. These annotations were classified as level 2 and/or level 3 according to the criteria suggested by Sumner et al. [76].

### Toxicity tests on *Daphnia magna*

The acute toxicity tests were carried out according to the guidelines of the Organization for Economic Cooperation and Development (OECD) [77], with some modifications. 20 neonate *D. magna* adults (coming from an established laboratory colony, > 20 generations, CRI, Czech Republic) were treated in each test vessel, and four replicates were performed for a total of 80 microcrustaceans per treatment group. *D. magna* adults were placed in 250 mL of pure water, the EO diluted in Tween 80 (Sigma Aldrich, Czech Republic) or the control (only Tween 80) were then emulsified into the water at concentrations ranging from 1 to 15 µL/L. *D. magna* mortality was evaluated using a stereomicroscope after 48 h from the treatment. The conditions for the assays were pH ranging from 7.2 to 7.6; 25 ± 1 °C; electrical conductivity around 160 μS/cm; dissolved oxygen above 3 mg/L and total hardness of 40–48 mg CaCO_3_/L.

### Statistical analysis

In mosquito toxicity tests, to quantify the toxicity of the different products, probit analysis was performed using the percentage of dead individuals after 24 h. The exposure concentrations (μg/mL) were log10 transformed, and the proportion of dead mosquitoes was used to calculate the median lethal concentration (LC_50_) and the lethal concentration required to kill 10, 30, and 90% of the individuals (LC_10_, LC_30_, and LC_90,_ respectively). Probit analyses were carried out with the “ecotox” R package [78] to estimate the LC_10_, LC_30_, LC_50_, and LC_90_ with the associated 95% confidence interval (CI) and Chi-squares. For each species, a Generalized Linear Mixed Model (GLMM) was fit to test the efficacy of products and different concentrations. As predictor variables, we used the percentage of mortality in terms of the number of dead mosquitoes on total samples, resulting in “1” when dead, and “0” for alive. A binomial distribution with replicate membership as a random effect was employed. Post hoc analysis was then performed using estimated marginal means with the Bonferroni correction to test which factors of the model—i.e., products and concentrations—had a significant effect on the dependent variable out and to examine the statistical differences between treatments and doses on the four species. Statistical analyses were carried out in R 4.3.1. [79]. In non-target experiments, *D. magna* mortality was adjusted with Abbott’s formula [80]. Then, LC_50_ and LC_90_ values and the associated 95% CI for each tested product were calculated using probit analysis [81] through the Biostat 5.9.8 software.

The metabolomic experiments were done in a completely randomized design with five replications. Before multivariate and univariate analysis, the MS-DIAL Extracted intensities were normalized on the internal standard (ribitol), log10 transformed, and Pareto scaled using the open-source software metaboanalyst 6.0 [82]. After data normalization, the multivariate unsupervised Principal component analysis (PCA) and the supervised orthogonal partial least-squares-discriminant analysis (OPLS-DA) were conducted to observe and visualize the metabolic changes across the different experimental groups. The Hotelling’s T2 region, represented as an ellipse in the model's score plots, defines the 95% confidence interval for the modeled variation. The Variable Importance in Projection (VIP) scores each variable's overall contribution to the OPLS-DA model, with variables having VIP scores greater than 1 deemed significant for group differentiation. To prevent overfitting, the OPLS-DA model was validated through a permutation test (using a permutation number equal to 20), considering the model valid if the empirical *p*-values for Q2 and R2Y were ≤ 0.05 and if Q2 and R2Y values were close to 1. Pairwise partial correlations between metabolites were computed using Debiased Sparse Partial Correlation (DSPC), with a degree cut-off equal to 2, to assess the relationship between two metabolites while controlling for the influence of all other related metabolites [83]. Successively, the data were analyzed through univariate analysis using the Student’s *t*-test (*P* ≤ 0.05), in which the *P* value was corrected by the False Discovery Rate (FDR ≤ 0.05), and the Fold Change analysis (FC), using an FC value of 1.5. Data were then graphically reported using a Volcano plot (corrected *P* value ≤ 0.05 and FC > 1.5). Data were finally analyzed through the enrichment and pathway analysis using the *Ae. aegypti* KEGG database. Concerning the cytotoxicity assay, the IC_50_ values were determined with GraphPad Prism 5 (San Diego, CA, USA) computer program.

## Supplementary Information

Additional file 1.

Additional file 2.

## Data Availability

All data generated or analysed during this study are included in this published article.

## Abbreviations

*C. myrrha*: *Commiphora myrrha*
*Ae. albopictus*: *Aedes albopictus*
*Ae. aegypti*: *Aedes aegypti*
*An. stephensi*: *Anopheles stephensi*
*An. gambiae*: *Anopheles gambiae*
*D. magna*: *Daphnia magna*
*C. caudata*: *Commiphora caudata*
*Cx. quinquefasciatus*: *Culex quinquefasciatus*
*C. berryi*: *Commiphora berryi*
*C. swynnertonii*: *Commiphora swynnertonii*
*C. erythraea*: *Commiphora erythraea*
*Cx. pipiens*: *Culex pipiens*
*Cx. restuans*: *Culex restuans*
*An. sinensis*: *Anopheles sinensis*
IC_50_: 50% Inhibition concentration
VBD: Vector-borne diseases
DMSO: Dimethyl sulfoxide
EtOH: Ethanol
LC_50_: 50% Lethal concentration
RI: Retention index
OECD: Organization for economic cooperation and development
oPLS-DA: Supervised orthogonal partial least-squares-discriminant analysis
VIP: Variable importance in projection
DSPC: Debiased sparse partial correlation
GABA: γ-Aminobutyric acid
GLMM: Generalized linear mixed model

## References

[1] Chala B, Hamde F. Emerging and re-emerging vector-borne infectious diseases and the challenges for control: a review. Front Public Health. 2021;9:1–10. 10.3389/fpubh.2021.715759. 10.3389/fpubh.2021.715759 PMC8524040 34676194

[2] Benelli G, Wilke AB, Beier JC. *Aedes albopictus* (Asian tiger mosquito). Trends Parasitol. 2020;36:942–3. 10.1016/j.pt.2020.01.001. 32037135 10.1016/j.pt.2020.01.001

[3] Pustijanac E, Buršić M, Millotti G, Paliaga P, Iveša N, Cvek M. Tick-borne bacterial diseases in Europe: threats to public health. Eur J Clin Microbiol Infect Dis. 2024. 10.1007/s10096-024-04836-5. 38676855 10.1007/s10096-024-04836-5

[4] Hemingway J, Ranson H. Insecticide resistance in insect vectors of human disease. Annu Rev Entomol. 2000;45:371–91. 10.1146/annurev.ento.45.1.371. 10761582 10.1146/annurev.ento.45.1.371

[5] Haddi K, Nauen R, Benelli G, Guedes RNC. Global perspectives on insecticide resistance in agriculture and public health. Entomol Gen. 2023;43:495–500. 10.1127/entomologia/2023/2186.

[6] Modafferi A, Giunti G, Benelli G, Campolo O. Ecological costs of botanical nano-insecticides. Curr Opin Environ Sci Health. 2024;42: 100579. 10.1016/j.coesh.2024.100579.

[7] Dolara P, Corte B, Ghelardini C, Pugliese AM, Cerbai E, Menichetti S, Nostro AL. Local anaesthetic, antibacterial and antifungal properties of sesquiterpenes from myrrh. Planta Med. 2000;66(04):356–8. 10.1055/s-2000-8532. 10865454 10.1055/s-2000-8532

[8] Abdul-Ghani RA, Loutfy N, Hassan A. Myrrh and trematodoses in Egypt: an overview of safety, efficacy and effectiveness profiles. Parasitol Internat. 2009;58(3):210–4. 10.1016/j.parint.2009.04.006. 10.1016/j.parint.2009.04.006 19446652

[9] Batiha GES, Wasef L, Teibo JO, Shaheen HM, Zakariya AM, Akinfe OA, Teibo TKA, Al-kuraishy HM, Al-Garbee AI, Alexiou A, Papadakis M. *Commiphora myrrh*: a phytochemical and pharmacological update. Naunyn-Schmiedeberg’s Arch Pharmacol. 2023;396(3):405–20. 10.1007/s00210-022-02325-0. 36399185 10.1007/s00210-022-02325-0 PMC9672555

[10] Leung AY, Foster S. Encyclopedia of common natural ingredients (used in food, drugs, and cosmetics). Hoboken: A John Wiley & Sons Inc.; 2003. p. 366–7.

[11] Lubbe A, Verpoorte R. Cultivation of medicinal and aromatic plants for specialty industrial materials. Ind Crops Prod. 2011;34(1):785–801. 10.1016/j.indcrop.2011.01.019.

[12] Nomicos EY. Myrrh: medical marvel or myth of the magi? Holist Nurs Pract. 2007;21(6):308–23. 10.1097/01.HNP.0000298616.32846.34. 17978635 10.1097/01.HNP.0000298616.32846.34

[13] Wahba TF, Aly HM, Hassan NA. The antifeedant properties of bio-oil from *Cupressus Sempervirens* against Rice Weevil (*Sitophilus oryzae*) compared to that of myrrh and frankincense oils. Egypt J Agric Res. 2023;101(2):331–41. 10.21608/ejar.2023.192485.1341.

[14] Zhu Y, Wu T, Hu Q, He W, Zheng Y, Xie Y, Rao Q, Liu X. Plant essential oils: dual action of toxicity and egg-laying inhibition on *Tetranychus urticae* (Acari:Tetranychidae), unveiling their potential as botanical pesticides. Plants. 2024;13(6):763. 10.3390/plants13060763. 38592755 10.3390/plants13060763 PMC10975855

[15] Dekebo A, Dagne E, Sterner O. Furanosesquiterpenes from *Commiphora sphaerocarpa* and related adulterants of true myrrh. Fitoterapia. 2002;73(1):48–55. 10.1016/S0367-326X(01)00367-7. 11864764 10.1016/s0367-326x(01)00367-7

[16] Marongiu B, Piras A, Porcedda S, Scorciapino A. Chemical composition of the essential oil and supercritical CO2 extract of *Commiphora myrrha* (Nees) Engl. and of *Acorus calamus* L. J Agric Food Chem. 2005;53(20):7939–43. 10.1021/jf051100x. 16190653 10.1021/jf051100x

[17] Morteza-Semnani K, Saeedi M. Constituents of the essential oil of *Commiphora myrrha* (Nees) Engl. Var. molmol. J Essent Oil Res. 2003;15(1):50–1. 10.1080/10412905.2003.9712264.

[18] Baldovini N, Tomi F, Casanova J. Identification and quantitative determination of furanodiene, a heat‐sensitive compound, in essential oil by ^13^C‐NMR. Phytochem Anal. 2001;12(1):58-63. 10.1002/1099-1565(200101/02)12:1<58::AID-PCA559>3.0.CO,2-9. 10.1002/1099-1565(200101/02)12:1<58::AID-PCA559>3.0.CO;2-9 11704963

[19] Van den Dool H, Krat PD. A generalization of the retention index system including linear temperature programmed gas-liquid partition chromatography. J Chromatogr. 1963;2:463–71. 10.1016/s0021-9673(01)80947-x 14062605

[20] Adams DRP. Identification of essential oil components by gas chromatography/. 2005.

[21] NIST N. Mass Spectral Library (NIST/EPA/NIH). 2005.

[22] Mondello L. FFNSC 2: flavors and fragrances of natural and synthetic compounds, mass spectral database. Software. 2011.

[23] Maggi F, Barboni L, Papa F, Caprioli G, Ricciutelli M, Sagratini G, Vittori S. A forgotten vegetable (*Smyrnium olusatrum* L., Apiaceae) as a rich source of isofuranodiene. Food Chem. 2012;135(4):2852–62. 10.1016/j.foodchem.2012.07.027. 22980882 10.1016/j.foodchem.2012.07.027

[24] https://jadebloom.com/media/wysiwyg/myrrh-gcms.pdf 2017. Accessed on 16 Aug 2024.

[25] Brieskorn CH, Noble P. Two furanoeudesmanes from the essential oil of myrrh. Phytochem. 1983;22(1):187–9. 10.1016/S0031-9422(00)80085-0.

[26] Pavela R, Pavoni L, Bonacucina G, Cespi M, Kavallieratos NG, Cappellacci L, Petrelli R, Maggi F, Benelli G. Rationale for developing novel mosquito larvicides based on isofuranodiene microemulsions. J Pest Sci. 2019;92:909–21. 10.1007/s10340-018-01076-3.

[27] Weyerstahl P, Marschall-Weyerstahl H, Christiansen C, Oguntimein BO, Adeoye AO. Volatile constituents of *Eugenia uniflora* leaf oil. Planta Med. 1988;54(6):546–9. 10.1055/s-2006-962544. 3212089 10.1055/s-2006-962544

[28] Alanazi NAH, Alamri AA, Mashlawi AM, Almuzaini N, Mohamed G, Salama SA. Gas chromatography-mass spectrometry chemical profiling of commiphora myrrha resin extracts and evaluation of larvicidal, antioxidant, and cytotoxic activities. Molecules. 2024;29:1778. 10.3390/molecules29081778. 38675598 10.3390/molecules29081778 PMC11051918

[29] Baranitharan M, Dhanasekaran S. Mosquito larvicidal properties of *Commiphora caudata* (Wight & Arn.) (Bursaceae) against *Aedes aegypti* (Linn.), *Anopheles stephensi* (Liston), *Culex quinquefasciatus* (Say). Int J Curr Microbiol App Sci. 2014;3:262–8.

[30] Baranitharan M, Dhanasekaran S, Gokulakrishnan J, Mahesh Babu S, Thushimenan S. Nagapattinam medicinal plants against the dengue fever mosquito, *Aedes aegypti*. Int J Mosq Res. 2016;3:29–34.

[31] Mkangara M, Chacha M, Kazyoba PE. Larvicidal potential of *Commiphora swynnertonii* (Burtt) stem bark extracts against *Anopheles gambiae* ss, *Culex quinquefasciatus* Say and *Aedes aegypti*. L Int J Sci Res. 2015;4:356–61.

[32] Muturi EJ, Hay WT, Doll KM, Ramirez JL, Selling G. Insecticidal activity of *Commiphora erythraea* essential oil and its emulsions against larvae of three mosquito species. J Med Entomol. 2020;57:1835–42. 10.1093/jme/tjaa097. 32474606 10.1093/jme/tjaa097

[33] Pavela R. Essential oils for the development of eco-friendly mosquito larvicides: a review. Ind Crops Prod. 2015;76:174–87. 10.1016/j.indcrop.2015.06.050.

[34] Costa JG, Pessoa OD, Menezes EA, Santiago GM, Lemos TL. Composition and larvicidal activity of essential oils from heartwood of *Auxemma glazioviana* Taub. (Boraginaceae). Flavour Fragr J. 2004;19(6):529–31. 10.1002/ffj.1332.

[35] Shaalan EAS, Canyon DV, Bowden B, Younes MWF, Abdel-Wahab H, Mansour AH. Efficacy of botanical extracts from *Callitris glaucophylla* against *Aedes aegypti* and *Culex annulirostris* mosquitoes. Trop Biomed. 2006;23:180–5. 17322820

[36] Triba MN, Le Moyec L, Amathieu R, Goossens C, Bouchemal N, Nahon P, Rutledge DN, Savarin P. PLS/OPLS models in metabolomics: the impact of permutation of dataset rows on the K-fold cross-validation quality parameters. Mol Biosyst. 2015;11(1):13–9. 10.1039/c4mb00414k. 25382277 10.1039/c4mb00414k

[37] Hu T, Zhang W, Fan Z, Sun G, Likhodi S, Randell E, Zhai G. Metabolomics differential correlation network analysis of osteoarthritis. In Biocomputing: Proceedings of the Pacific Symposium. World Scientific Publishing Company. Singapore; 2016. pp. 120–131. 26776179

[38] Chong J, Soufan O, Li C, Caraus I, Li S, Bourque G, Wishart DS, Xia J. MetaboAnalyst 4.0: towards more transparent and integrative metabolomics analysis. Nucleic Acids Res. 2018;46(W1):W486–94. 10.1093/nar/gky310. 29762782 10.1093/nar/gky310 PMC6030889

[39] Brinzer RA, Henderson L, Marchiondo AA, Woods DJ, Davies SA, Dow JA. Metabolomic profiling of permethrin-treated *Drosophila melanogaster* identifies a role for tryptophan catabolism in insecticide survival. Insect Biochem Mol Biol. 2015;67:74–86. 10.1016/j.ibmb.2015.09.009. 26474926 10.1016/j.ibmb.2015.09.009

[40] Gao YP, Luo M, Wang XY, He XZ, Lu W, Zheng XL. Pathogenicity of *Beauveria bassiana* PfBb and immune responses of a non-target host, *Spodoptera frugiperda* (Lepidoptera: Noctuidae). Insects. 2022;13(10):914. 10.3390/insects13100914. 36292862 10.3390/insects13100914 PMC9604019

[41] Cerstiaens A, Huybrechts J, Kotanen S, Lebeau I, Meylaers K, De Loof A, Schoofs L. Neurotoxic and neurobehavioral effects of kynurenines in adult insects. Biochem Biophys Res Commun. 2003;312(4):1171–7. 10.1016/j.bbrc.2003.11.051. 14651996 10.1016/j.bbrc.2003.11.051

[42] Li Y, Li Y, Wang G, Li J, Zhang M, Wu J, Liang C, Zhou H, Tang J, Zhu G. Differential metabolome responses to deltamethrin between resistant and susceptible *Anopheles sinensis*. Ecotoxicol Env Saf. 2022;237:113553. 10.1016/j.ecoenv.2022.113553. 35483147 10.1016/j.ecoenv.2022.113553

[43] Mansingh A. The effect of malathion on the metabolism of amino acids in the German cockroach *Blattella germanica*. J Insect Physiol. 1965;11(10):1389–400. 10.1016/0022-1910(65)90176-9. 5829268 10.1016/0022-1910(65)90176-9

[44] Rand EED, Smit S, Beukes M, Apostolides Z, Pirk CW, Nicolson SW. Detoxification mechanisms of honeybees (*Apis mellifera*) resulting in tolerance of dietary nicotine. Sci Rep. 2015;5(1):11779. 10.1038/srep11779. 26134631 10.1038/srep11779 PMC4488760

[45] Marco L, Sassera D, Epis S, Mastrantonio V, Ferrari M, Ricci I, Comandatore F, Bandi C, Porretta D, Urbanelli S. The choreography of the chemical defensome response to insecticide stress: insights into the *Anopheles stephensi* transcriptome using rna-seq. Sci Rep. 2017;7(1):1312. 10.1038/srep41312. 28112252 10.1038/srep41312 PMC5256098

[46] Mack L. Time-series analysis of transcriptomic changes due to permethrin exposure reveals that *Aedes aegypti* undergoes detoxification metabolism over 24 h. Sci Rep. 2023;13(1):16564. 10.1038/s41598-023-43676-9. 37783800 10.1038/s41598-023-43676-9 PMC10545687

[47] Zhang C, Yuan H, Hu Y, Li X, Gao Y, Ma Z, Lei P. Structural diversity design, synthesis, and insecticidal activity analysis of ester-containing isoxazoline derivatives acting on the GABA receptor. J Agric Food Chem. 2023;71(7):3184–91. 10.1021/acs.jafc.2c07910. 10.1021/acs.jafc.2c07910 36757129

[48] Homberg U, Humberg T, Seyfarth J, Bode K, Pérez M. GABA immunostaining in the central complex of dicondylian insects. J Comp Neurol. 2018;526(14):2301–18. 10.1002/cne.24497. 30004590 10.1002/cne.24497

[49] Zulfiqar F, Akram N, Ashraf M. Osmoprotection in plants under abiotic stresses: new insights into a classical phenomenon. Planta. 2019. 10.1007/s00425-019-03293-1. 31776765 10.1007/s00425-019-03293-1

[50] Darkó É, Végh B, Khalil R, Marček T, Szalai G, Pál M, Janda T. Metabolic responses of wheat seedlings to osmotic stress induced by various osmolytes under iso-osmotic conditions. PLoS ONE. 2019;14(12): e0226151. 10.1371/journal.pone.0226151. 31856179 10.1371/journal.pone.0226151 PMC6922385

[51] USEPA, United States Environmental Protection Agency. Methods for Measuring the Acute Toxicity of Effluent to Freshwater and Marine Organisms. 3rd ed. USEPA. United States Environmental Protection Agency. Washington, DC, United States; 1985.

[52] FATMA, Limites Máximos de Toxidade Aguda para efluentes de diferentes origins. In: PORTARIA No. 017/02; 2002.

[53] Ural MS, Saglam N. A study on the acute toxicity of pyrethroid deltamethrin on the fry rainbow trout (*Oncorhynchus mykiss* Walbaum, 1792). Pestic Biochem Physiol. 2005;83(2–3):124–31. 10.1016/j.pestbp.2005.04.004.

[54] Başer S, Erkoç F, Selvi M, Koçak O. Investigation of acute toxicity of permethrin on guppies *Poecilia reticulata*. Chemosphere. 2003;51(6):469–74. 10.1016/S0045-6535(03)00033-X. 12615098 10.1016/S0045-6535(03)00033-X

[55] Barata C, Baird DJ, Nogueira AJA, Soares AMVM, Riva MC. Toxicity of binary mixtures of metals and pyrethroid insecticides to *Daphnia magna* Straus. Implications for multi-substance risks assessment. Aquat Toxicol. 2006;78(1):1–14. 10.1016/j.aquatox.2006.01.013. 16510198 10.1016/j.aquatox.2006.01.013

[56] Imgrund H. Environmental Fate of Permethrin. In Environmental Monitoring Branch. Department of Pesticide Regulation. Sacramento, CA, USA; 2003.

[57] Maranho LA, Botelho RG, Mitie Inafuku M, Nogueira L, de Olinda AR, InaciodeSousa BAI, Tornisielo VL. Testing the neem biopesticide (*Azadirachta indica* A. Juss) for acute toxicity with *Danio rerio* and for chronic toxicity with Daphnia magna. J Agric Sci Tech. 2014;16(1):105–11.

[58] Ulrich J, Stiltz S, St-Gelais A, El Gaafary M, Simmet T, Syrovets T, Schmiech M. Phytochemical composition of *Commiphora* oleogum resins and their cytotoxicity against skin cancer cells. Molecules. 2022;27(12):3903. 10.3390/molecules27123903. 35745024 10.3390/molecules27123903 PMC9229828

[59] Sun X-Y, Zheng Y-P, Lin D-H, Zhang H, Zhao F, Yuan C-S. Potential anti-cancer activities of furanodiene, a sesquiterpene from *Curcuma wenyujin*. Am J Chin Med. 2009;37(03):589–96. 10.1142/S0192415X09007077. 19606517 10.1142/S0192415X09007077

[60] Quassinti L, Bramucci M, Lupidi G, Barboni L, Ricciutelli M, Sagratini G, Papa F, Caprioli G, Petrelli D, Vitali LA, Vittori S, Maggi F. In Vitro biological activity of essential oils and isolated furanosesquiterpenes from the neglected vegetable *Smyrnium olusatrum* L. (Apiaceae). Food Chem. 2013;138(2):808–13. 10.1016/j.foodchem.2012.11.075. 23411181 10.1016/j.foodchem.2012.11.075

[61] Wang C-C, Chen L-G, Yang L-L. Cytotoxic activity of sesquiterpenoids from *Atractylodes ovata* on Leukemia cell lines. Planta Med. 2002;68(3):204–8. 10.1055/s-2002-23144. 11914954 10.1055/s-2002-23144

[62] Wang K-T, Chen L-G, Yang L-L, Ke W-M, Chang H-C, Wan C-C. Analysis of the sesquiterpenoids in processed *Atractylodis* rhizoma. Chem Pharm Bull. 2007;55(1):50–6. 10.1248/cpb.55.50. 10.1248/cpb.55.50 17202701

[63] Spinozzi E, Ferrati M, Baldassarri C, Petrelli R, Cappellacci L, De Fazi L, Benelli G, Maggi F. Unlocking the potential of alexanders (*Smyrnium olusatrum* L., Apiaceae): a neglected species with future crop prospect. Ind Crops Prod. 2024;218:118847. 10.1016/j.indcrop.2024.118847.

[64] Wang Y, Li J, Guo J, Wang Q, Zhu S, Gao S, Yang C, Wei M, Pan X, Zhu W, Ding D, Gao R, Zhang W, Wang J, Zang L. Cytotoxic and antitumor effects of curzerene from *Curcuma longa*. Planta Med. 2016;83(01/02):23–9. 10.1055/s-0042-107083. 27286338 10.1055/s-0042-107083

[65] Pavela R, Pavoni L, Bonacucina G, Cespi M, Cappellacci L, Petrelli R, Spinozzi E, Aguzzi C, Zeppa L, Ubaldi M, Desneux N, Canale A, Maggi F, Benelli G. Encapsulation of *Carlina acaulis* essential oil and carlina oxide to develop long-lasting mosquito larvicides: Microemulsions versus nanoemulsions. J Pest Sci. 2021;94:899–915. 10.1007/s10340-020-01327-2.

[66] Stepanenko AA, Dmitrenko VV. HEK293 in cell biology and cancer research: phenotype, karyotype, tumorigenicity, and stress-induced genome-phenotype evolution. Gene. 2015;569(2):182–90. 10.1016/j.gene.2015.05.065. 26026906 10.1016/j.gene.2015.05.065

[67] Gugliuzzo A, Francardi V, Simoni S, Roversi PF, Ferrati M, Spinozzi E, Perinelli DR, Bonacucina G, Maggi F, Tortorici S, Tropea Garzia G, Biondi A, Rizzo R. Role of plant essential oil nanoemulsions on host colonization by the invasive ambrosia beetle *Xylosandrus compactus*. Ind Crops Prod. 2023;195: 116437. 10.1016/j.indcrop.2023.116437.

[68] Maggi F, Papa F, Giuliani C, Maleci Bino L, Venditti A, Bianco A, Nicoletti M, Iannarelli R, Caprioli G, Sagratini G, Cortese M, Ricciutelli M, Vittori S. Essential oil chemotypification and secretory structures of the neglected vegetable *Smyrnium olusatrum* L. (Apiaceae) growing in central Italy. Flavour Fragr J. 2015;30(2):139–59. 10.1002/ffj.3221.

[69] Williams CM, Mander LN. Chromatography with silver nitrate. Tetrahedron. 2001;57(3):425–47. 10.1016/S0040-4020(00)00927-3.

[70] Mander LN, Williams CM. Chromatography with silver nitrate: part 2. Tetrahedron. 2016;72(9):1133–50. 10.1016/j.tet.2016.01.004.

[71] Damiens D, Benedict M, Wille M, Gilles J. An inexpensive and effective larval diet for *Anopheles arabiensis* (Diptera: Culicidae): eat like a horse, a bird, or a fish? J Med Entomol. 2012;49:1001–11. 10.1603/ME11289. 23025180 10.1603/me11289

[72] WHO. Report of the WHO Informal Consultation on the Evaluation and Testing of 854 Insecticides. WHO Geneva. 1996;10:1026–1032.

[73] Page M, Bejaoui N, Cinq-Mars B, Lemieux P. Optimization of the tetrazoliun-based colorimetric assay for the measurement of cell number and cytotoxicity. Int J Immunopharmacol. 1998;10(7):785–93. 10.1016/0192-0561(88)90001-X. 10.1016/0192-0561(88)90001-x 3235236

[74] Misra BB, Das V, Landi M, Abenavoli MR, Araniti F. Short-term effects of the allelochemical umbelliferone on *Triticum durum* L. metabolism through GC–MS based untargeted metabolomics. Plant Sci. 2020;298:110548. 10.1016/j.plantsci.2020.110548. 32771160 10.1016/j.plantsci.2020.110548

[75] Misra B. Steps for building an open source EI-MS mass spectral library for GC-MS -based metabolomics. Metabolomics Protocols & Workflows. 2019; 10.17504/protocols.io.8txhwpn

[76] Sumner LW, Amberg A, Barrett D, Beale MH, Beger R, Daykin CA, Fan W-MT, Fiehn O, Goodacre R, Griffin JL, Hankemeier T, Hardy N, Harnly J, Higashi R, Kopka J, Lane AN, Lindon JC, Marriott P, Nicholls AW, Reily MD, Thaden JJ, Viant MR. Proposed minimum reporting standards for chemical analysis: chemical analysis working group (CAWG) metabolomics standards initiative (MSI). Metabolomics. 2007;3:211–21. 10.1007/s11306-007-0082-2. 24039616 10.1007/s11306-007-0082-2 PMC3772505

[77] OECD. Guideline for testing of chemicals. *Daphnia* sp., acute immobilisation test. 2004.

[78] Hlina BL, Birceanu O, Robinson CS, Dhiyebi H, Wilkie MP. The relationship between thermal physiology and lampricide sensitivity in larval sea lamprey (*Petromyzon marinus*). J Great Lakes Res. 2021;47:S272–84. 10.1016/j.jglr.2021.10.002.

[79] R Development Core Team. R: A Language and Environment for Statistical Computing. R Foundation for Statistical Computing. 2008. http://www.R-project.org. Accessed 26 Jul 2024.

[80] Abbott WS. A method of computing the effectiveness of an insecticide. J Econ Entomol. 1925;18(2):265–7.

[81] Finney DJ. Probit analysis. London: Cambridge University Press; 1971.

[82] Pang Z, Lu Y, Zhou G, Hui F, Xu L, Viau C, Spigelman AF, MacDonald PE, Wishart DS, Li S, Xia J. MetaboAnalyst 6.0: towards a unified platform for metabolomics data processing, analysis and interpretation. Nucleic Acids Res. 2024;52:W398–406. 10.1093/nar/gkae253. 38587201 10.1093/nar/gkae253 PMC11223798

[83] Basu S, Duren W, Evans CR, Burant CF, Michailidis G, Karnovsky A. Sparse network modeling and metscape-based visualization methods for the analysis of large-scale metabolomics data. Bioinformatics. 2017;33(10):1545–53. 10.1093/bioinformatics/btx012. 28137712 10.1093/bioinformatics/btx012 PMC5860222

