# Supplementary material for: Essential oil and furanosesquiterpenes from myrrh oleo-gum resin: a breakthrough in mosquito vector management
Source: Nat Prod Bioprospect. 2025 Jan 20;15(1):12. doi: 10.1007/s13659-024-00492-6 (PMC11753448; doi:10.1007/s13659-024-00492-6)
Supplement: Supplementary file 1 — Additional file 1. [file 13659_2024_492_MOESM1_ESM.docx]

**SUPPORTING INFORMATION**

**Essential oil and furanosesquiterpenes from myrrh oleo-gum resin: a breakthrough in mosquito vector management**

Eleonora Spinozzi^1*†^, Marta Ferrati^1†^, Cecilia Baldassarri^2^, Paolo Rossi^2^, Guido Favia^2^, Giorgio Cameli^1^, Giovanni Benelli^3^, Angelo Canale^3^, Livia De Fazi^3^, Roman Pavela^4,5^, Luana Quassinti^6^, Cristiano Giordani^7,8^, Fabrizio Araniti^9^, Loredana Cappellacci^1^, Riccardo Petrelli^1^, Filippo Maggi^1^

^1^ Chemistry Interdisciplinary Project (ChIP) research center, School of Pharmacy, University of Camerino, Via Madonna delle Carceri, 62032 Camerino, Italy. [eleonora.spinozzi@unicam.it](mailto:Eleonora.spinozzi@unicam.it); [marta.ferrati@unicam.it](mailto:marta.ferrati@unicam.it); [giorgio.cameli@studenti.unicam.it](mailto:giorgio.cameli@studenti.unicam.it); [loredana.cappellacci@unicam.it](mailto:loredana.cappellacci@unicam.it); [riccardo.petrelli@unicam.it](mailto:riccardo.petrelli@unicam.it); [filippo.maggi@unicam.it](mailto:filippo.maggi@unicam.it)

^2^ School of Bioscience and Veterinary Medicine, University of Camerino, Via Gentile III Da Varano, 62032 Camerino, Italy. [cecilia.baldassarri@unicam.it](mailto:cecilia.baldassarri@unicam.it); [paolo.rossi@unicam.it](mailto:paolo.rossi@unicam.it); [guido.favia@unicam.it](mailto:guido.favia@unicam.it)

^3^ Department of Agriculture, Food and Environment, University of Pisa, Via del Borghetto 80, 56124, Pisa, Italy. [giovanni.benelli@unipi.it](mailto:giovanni.benelli@unipi.it); angelo.canale@unipi.it; [livia.defazi@phd.unipi.it](mailto:livia.defazi@phd.unipi.it)

^4^ Crop Research Institute, Drnovska 507, 161 06 Prague, Czech Republic. [pavela@vurv.cz](mailto:pavela@vurv.cz)

^5^ Department of Plant Biotechnology, College of Life Sciences and Biotechnology, Korea University, Seoul 02841, Republic of Korea

^6^ School of Pharmacy, University of Camerino, Camerino, Italy. [luana.quassinti@unicam.it](mailto:luana.quassinti@unicam.it)

^7^ Instituto de Física, Universidad de Antioquia, UdeA, Calle 70 No 52-21, Medellín 050010, Colombia. [cristiano.giordani@udea.edu.co](mailto:cristiano.giordani@udea.edu.co)

^8^ Grupo Productos Naturales Marinos, Facultad de Ciencias Farmacéuticas y Alimentarias, Universidad de Antioquia, Calle 70 No. 52-21, Medellín 050010, Colombia. [cristiano.giordani@udea.edu.co](mailto:cristiano.giordani@udea.edu.co)

^9^ Dipartimento di Scienze Agrarie e Ambientali - Produzione, Territorio, Agroenergia, Università Statale di Milano, Via Celoria n. 2, 20133, Milan, Italy. [fabrizio.araniti@unimi.it](mailto:fabrizio.araniti@unimi.it)

^†^ These authors contributed equally.

* Corresponding author: Chemistry Interdisciplinary Project (ChIP) research center, School of Pharmacy, University of Camerino, Camerino, Italy. E-mail address: [eleonora.spinozzi@unicam.it](mailto:eleonora.spinozzi@unicam.it)

**1 Material and methods**

**1.1 HPLC-DAD quantitative analysis**

Individual stock solutions of furanoeudesma-1,3-diene, isofuranodiene, and curzerene were prepared by dissolving each compound into 10 mL of acetonitrile. The calibration curves were constructed by injecting different dilutions of the stock solutions as follows: 158.8-1984.5 μg/mL for furanoeudesma-1,3-diene, 15.0-1440.0 μg/mL for isofuranodiene, and 14.3-1111.2 μg/mL for curzerene (Fig. S1). Each solution was filtered through a disposable Minisart SRP4 filter of 0.45 μm (Chromaﬁl PET-20/25) (Sartorius Stedim Biotech GmbH, Goettingn, Germany).


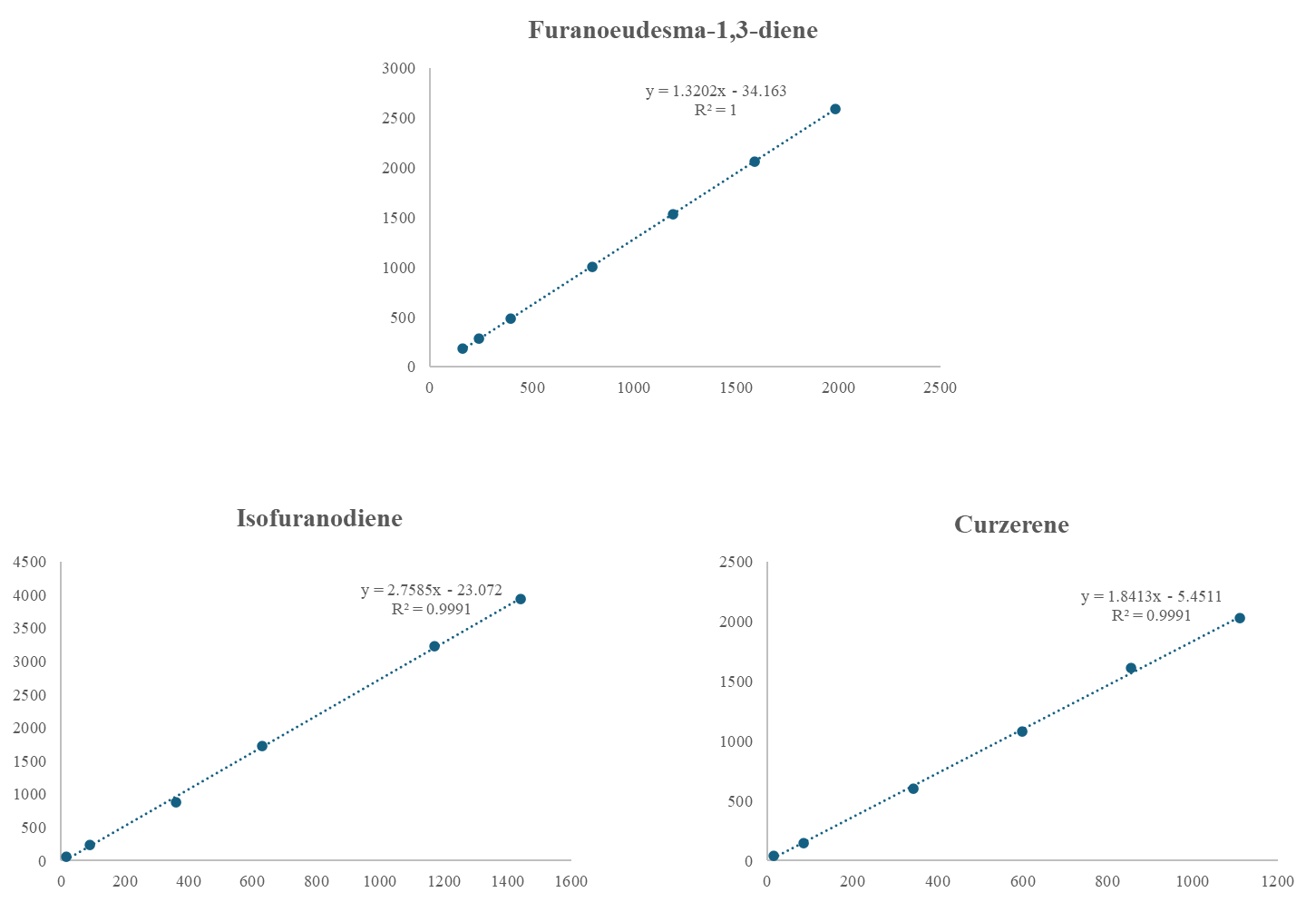


**Fig. S1** Calibration curves of furanoeudesma-1,3-diene, isofuranodiene, and curzerene

The analytical method was validated in terms of linearity, repeatability, limits of detection (LODs), and limits of quantification (LOQs). The reproducibility was assessed by determining the relative standard deviation (%RSD) between consecutive analyses (n = 5) performed the same day (intraday reproducibility) and during 5 consecutive days (interday reproducibility). LOD and LOQ were experimentally calculated by injecting low concentrations of the analytes standard solutions and measuring the signal-to noise (S/N) ratio. A concentration giving a S/N ratio (height of peak/ height of noise) of 3 was assigned to LOD while that of 10 was LOQ (Table S1).

**Table S1** HPLC-DAD method validation

| **Compound** | **Conc. range**  **(μg/mL)** | **Regression equation^a^** | **R^2b^** | **LOD (μg/mL)^c^** | **LOQ (μg/mL)^d^** | **Reproducibility (%RSD)^e^** | |
| --- | --- | --- | --- | --- | --- | --- | --- |
|  |  |  |  |  |  | **Intraday^f^** | **Interday^g^** |
| Furanoeudesma-1,3-diene | 158.8-1984.5 | *y = 1.3202x - 34.163* | 1 | 0.52 | 1.75 | 0.36 | 5.44 |
| Isofuranodiene | 15.0-1440.0 | *y = 2.7585x - 23.072* | 0.9991 | 0.13 | 0.42 | 0.87 | 0.98 |
| Curzerene | 14.3-1111.2 | *y = 1.8413x - 5.4511* | 0.9991 | 0.91 | 3.02 | 1.27 | 1.56 |

^a^y=concentration (μg/mL), x=analyte peak area/internal standard peak area; ^b^R^2^, coefficient of determination; ^c^LOD, limit of detection (3 × signal-to-noise (S/N)); ^d^LOQ, limit of quantification (10 × signal-to-noise (S/N) ratio); ^e^RSD, relative standard deviation; ^f^ intraday reproducibility, obtained by analyzing 3 times a mixture standard solution on the same day; ^g^interday reproducibility, obtained by analyzing standard solutions during 3 consecutive days

**1.2 Purification of furanosesquiterpenes**

For the purification of the furanosesquiterpenes, silica was absorbed with a 20% solution of AgNO_3_ (1:1 w/w) in acetonitrile. After the evaporation of the solvent, the silica was left to dry in oven at 35°C for 90 min. Fraction 2 was loaded into the column through dry charge on silica absorbed with the 20% AgNO_3_ solution. The mobile phase consisted of *n*-hexane/ethyl acetate (98:2) and the polarity was gradually increased during the purification up to *n*-hexane/ethyl acetate (95:5). The separation was monitored using TLC plates absorbed with a 20% solution of AgNO_3_ [1]_._ In detail, the TLC were left soaking in 10 mL of the above-mentioned solution for 10 minutes and they were left drying until complete evaporation of the solvent. Then, the separation of the compounds was verified by oxidizing the TLC with a cerium molybdate stain.

**1.3 MTT cytotoxicity assay**

Briefly, the human embryonic kidney 293 cell line (HEK293) was cultured in Eagle’s Minimum Essential Medium (MEM) with 2 mM L-glutamine, 100 IU/mL penicillin, 100 μg/mL streptomycin, and supplemented with 10% heat-inactivated fetal bovine serum (HI-FBS). Cells were maintained in a humidified atmosphere at 37°C in the presence of 5% CO_2_. Cells were seeded at the density of 2 x 10^4^ cells/mL. After 24 h, samples were exposed to different concentrations of EO (0.78-100 μg/mL) in EtOH. The anticancer drug cisplatin (0.01–50 μg/mL) was used as the positive control. Cells were incubated for 72 h in a humidified atmosphere of 5% CO_2_ at 37°C. At the end of incubation, 10 µL of MTT (5 μg/mL in phosphate-buffered saline, PBS) per well were added and the plates were incubated for 4 h at 37°C. Then the supernatant was removed, and DMSO was added to dissolve the formazan crystals. The extent of MTT reduction was measured spectrophotometrically at 540 nm using a microplate spectrophotometer FLUOstar Omega (BMG Labtech, Milan, Italy). The cell survival curves were calculated after the comparison with those of cells exposed to the vehicle (EtOH).

**2 Results**

**2.1 GC-MS analysis of the essential oil (EO) and its fractions**

**Table S2** Chemical composition of fraction 1 of Commiphora myrrha essential oil (EO)

| **No.** | **Compound^a^** | **Chemical class^b^** | **RI^c^** | **RI Lit^d^** | **Area% ± SD^e^** | **Id.^f^** |
| --- | --- | --- | --- | --- | --- | --- |
| 1 | (*E*)-*β*-ocimene | MH | 1048 | 1044 | 0.28 ± 0.00 | RI, MS |
| 2 | *δ*-elemene | SH | 1337 | 1335 | 6.94 ± 0.07 | RI, MS |
| 3 | *β*-bourbonene | SH | 1384 | 1387 | 2.53 ± 0.03 | RI, MS |
| 4 | *β*-elemene | SH | 1392 | 1389 | 29.08 ± 0.70 | RI, MS |
| 5 | (*E*)-caryophyllene | SH | 1418 | 1418 | 4.27 ± 0.01 | RI, MS |
| 6 | *γ*-elemene | SH | 1433 | 1434 | 5.64 ± 0.02 | RI, MS |
| 7 | *α*-humulene | SH | 1452 | 1452 | 1.56 ± 0.00 | RI, MS |
| 8 | *γ*-muurolene | SH | 1476 | 1478 | 2.55 ± 0.06 | RI, MS |
| 9 | germacrene D | SH | 1480 | 1484 | 4.79 ± 0.07 | RI, MS |
| 10 | *β*-selinene | SH | 1485 | 1489 | 6.33 ± 0.04 | RI, MS |
| 11 | *α*-selinene | SH | 1494 | 1498 | 6.12 ± 0.01 | RI, MS |
| 12 | *α*-muurolene | SH | 1499 | 1500 | 0.79 ± 0.04 | RI, MS |
| 13 | (*Z*)-*α*-bisabolene | SH | 1503 | 1506 | 0.85 ± 0.03 | RI, MS |
| 14 | *γ*-cadinene | SH | 1513 | 1513 | 2.42 ± 0.05 | RI, MS |
| 15 | *δ*-cadinene | SH | 1523 | 1522 | 3.15 ± 0.06 | RI, MS |
| 16 | selina-3,7-(11)-diene | SH | 1541 | 1545 | 1.28 ± 0.09 | RI, MS |
| 17 | germacrene B | SH | 1556 | 1549 | 15.81 ± 0.01 | RI, MS |
|  | Total identified |  |  |  | 94.37 ± 0.27 |  |
|  | Monoterpene hydrocarbons (MH) |  |  |  | 0.28 |  |
|  | Sesquiterpene hydrocarbons (SH) |  |  |  | 94.09 |  |

^a^Compounds are listed according to their order of elution from the HP-5MS column; ^b^Chemical class: MH, monoterpenes hydrocarbons; SH, sesquiterpenes hydrocarbons; ^c^Linear retention index calculated with the Van den Dool and Kratz (1963) formula; ^d^Retention index from Adams (2007); ^e^Relative percentage values derived from two independent analyses. SD, standard deviation; ^f^Method of identification: Std, comparison with available analytical standards; RI, linear with those calculated with ADAMS (2007) and NIST (2020) libraries; MS, correspondence of the mass spectrum with respect to that of ADAMS (2007), FFNSC (2012), and NIST20 libraries

**Table S3** Chemical composition of fraction 2 of Commiphora myrrha essential oil (EO)

| **No.** | **Compound^a^** | **Chemical class^b^** | **RI^c^** | **RI Lit^d^** | **Area% ± SD^e^** | **Id.^f^** |
| --- | --- | --- | --- | --- | --- | --- |
| 1 | curzerene | FS | 1497 | 1499 | 33.90 ± 0.24 | Std, RI, MS |
| 2 | furanoeudesma-1,3-diene | FS | 1626 | - | 44.95 ± 0.05 | Std, MS |
| 3 | lindestrene | FS | 1632 | 1623^g^ | 16.67 ± 0.15 | RI, MS |
| 4 | unknown furanosesquiterpene^i^ | FS | 1656 | - | 0.72 ± 0.03 | MS |
| 5 | atractylon | FS | 1660 | 1657 | 1.60 ± 0.04 | RI, MS |
| 6 | isofuranodiene | FS | 1691 | 1688^h^ | 1.85 ± 0.07 | Std, RI, MS |
|  |  |  |  |  |  |  |
|  | Total identified |  |  |  | 99.70 ± 0.01 |  |

^a^Compounds are listed accordingly to their order of elution from the HP-5MS column; ^b^Chemical class: FS, furanosesquiterpene; ^c^Linear retention index calculated with the Van den Dool and Kratz (1963) formula; ^d^Retention index from Adams (2007); ^e^Relative percentage values derived from two independent analyses. SD, standard deviation; ^f^Methods of identification: Std, comparison with available analytical standards; RI, linear with those calculated with ADAMS (2007) and NIST (2020) libraries; MS, correspondence of the mass spectrum with respect to that of ADAMS (2007), FFNSC (2012), and NIST20 libraries; ^g,h^RI comparable with literature (Maggi et al., 2012; https://phytochemia.com/); ^i^MS (EI): m/z = 216 (M^+^), 145, 121, 108, 91, 79, 44

**2.2 Purification of furanosesquiterpenes**

**Furanoeudesma-1,3-diene:** white solid; racemic mixture. Purity > 95%, determined by GC-MS and NMR analyses. IR (neat) 2990, 2910, 2833, 1652, 1467, 1411, 1120, 748 cm^-1^; ^1^H NMR (500 MHz, CDCl_3_) *δ* 7.08 (t, *J* = 1.4 Hz, 1H), 5.80 (dd, *J* = 9.4, 5.2 Hz, 1H), 5.73 – 5.69 (m, 1H), 5.54 (dt, *J* = 9.6, 1.1 Hz, 1H), 2.74 (dt, *J* = 16.0, 2.3 Hz, 1H), 2.69 – 2.62 (m, 1H), 2.54 (m, 1H), 2.42 (dd, *J* = 16.0, 1.6 Hz, 1H), 2.29 (ddd, *J* = 15.6, 12.4, 3.3, 1.6 Hz, 1H), 1.96 (s, 3H), 1.88 (s, 3H), 0.84 (s, 3H); ^13^C NMR (125 MHz, CDCl_3_) *δ* 150.28, 137.90, 137.36, 135.99, 122.45, 120.38, 119.88, 117.09, 43.26, 37.02, 35.68, 20.77, 20.15, 15.74, 8.50; MS (EI): *m/z* = 214 (M^+^), 199, 118, 108, 91,79; Anal. calcd. for (C_15_H_18_O) C, 84.07; H, 8.47; Found: C, 84.03; H, 8.49. The NMR spectra are reported in Fig. S2, S3, and S4.

**Isofuranodiene**: white solid; racemic mixture. Purity > 95%, determined by GC-MS and NMR analyses. IR (neat) 2964, 2924, 2861, 1664, 1437, 1386, 1138, 754 cm^-1^; ^1^H NMR (500 MHz, CDCl_3_) *δ* 7.06 (s, 1H), 4.86 (q, *J* = 1.8 Hz, 1H), 4.72 – 4.68 (m, 1H), 2.47 – 2.33 (m, 4H), 1.95 (t, *J* = 1.7 Hz, 3H), 1.76 – 1.63 (m, 2H), 1.61 – 1.48 (m, 2H), 0.76 (s, 6H); ^13^C NMR (125 MHz, CDCl_3_) *δ* 137.31, 134.79, 122.27, 119.97, 119.92, 116.51, 107.61, 39.68, 39.09, 33.57, 23.95, 21.26, 17.91, 8.49. MS (EI): *m/z* = 216 (M^+^), 159, 145, 108, 93; Anal. calcd. for (C_15_H_20_O) C, 83.28; H, 9.32; Found: C, 83.25; H, 9.34.

**Curzerene**: colorless oil; racemic mixture. Purity > 95%, determined by GC-MS and NMR analyses. IR (neat) 3082, 2968, 2908, 1637, 1441, 1414, 1112, 734 cm^-1^; ^1^H NMR (500 MHz, CDCl_3_) *δ* 7.06 (s, 1H), 5.88 (m, 1H), 5.00 – 4.94 (m, 2H), 4.87 (m, 1H), 4.75 (s, 1H), 2.71 – 2.64 (m, 2H), 2.42 (d, *J* = 6.6 Hz, 3H), 1.92 (s, 3H), 1.74 (s, 3H), 1.07 (s, 3H). MS (EI): *m/z* = 216 (M^+^), 148, 108, 201, 79; ^13^C NMR (125 MHz, CDCl_3_) *δ* 149.88, 147.58, 147.52, 137.54, 119.68, 116.86, 113.08, 111.32, 50.37, 40.48, 36.46, 24.77, 24.55, 19.96, 8.43; Anal. calcd. for (C_15_H_20_O) C, 83.28; H, 9.32; Found: C, 83.24; H, 9.31.


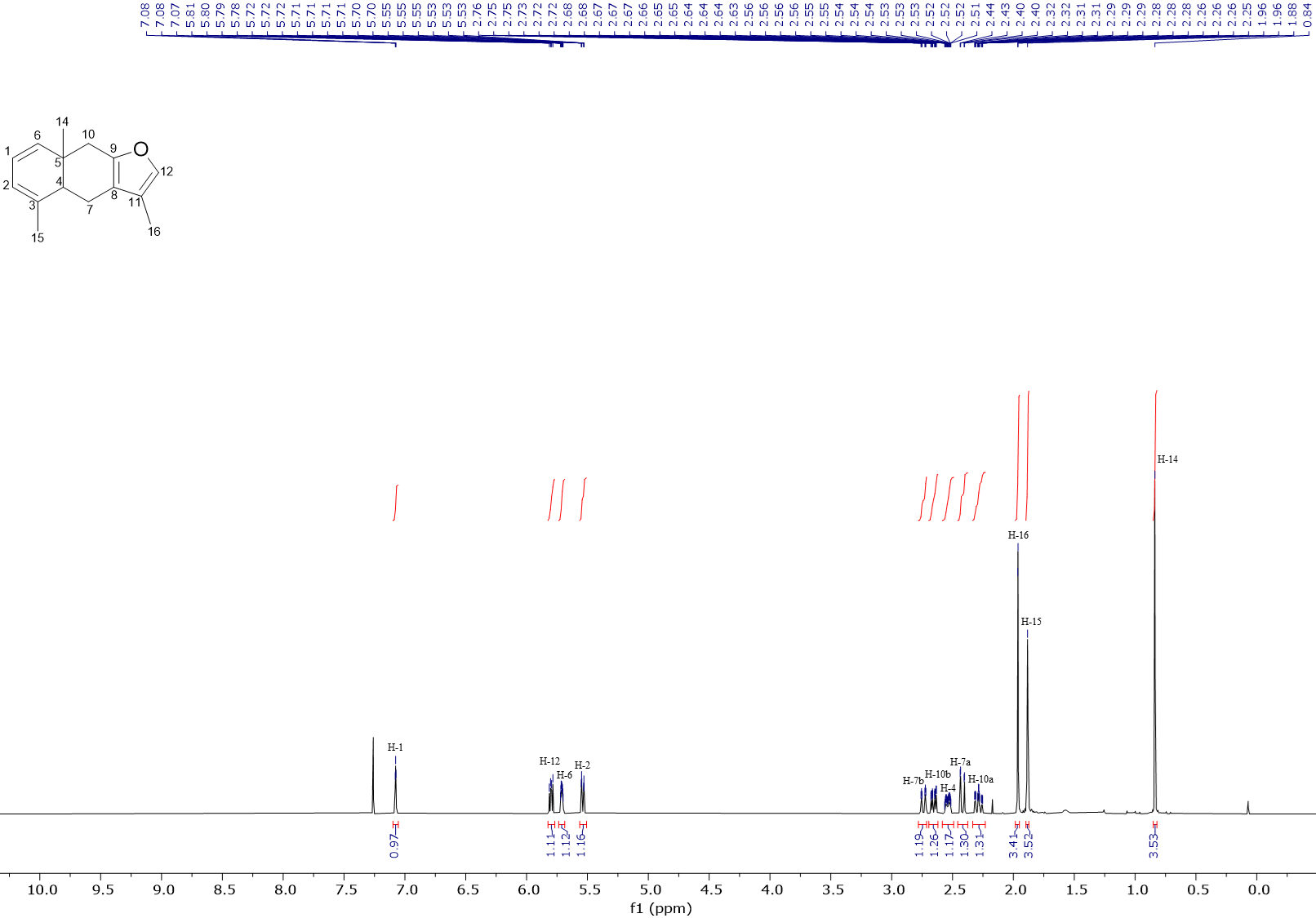


**Fig. S2** ^1^H NMR spectrum of furanoeudesma-1,3-diene (in CDCl_3_)

**
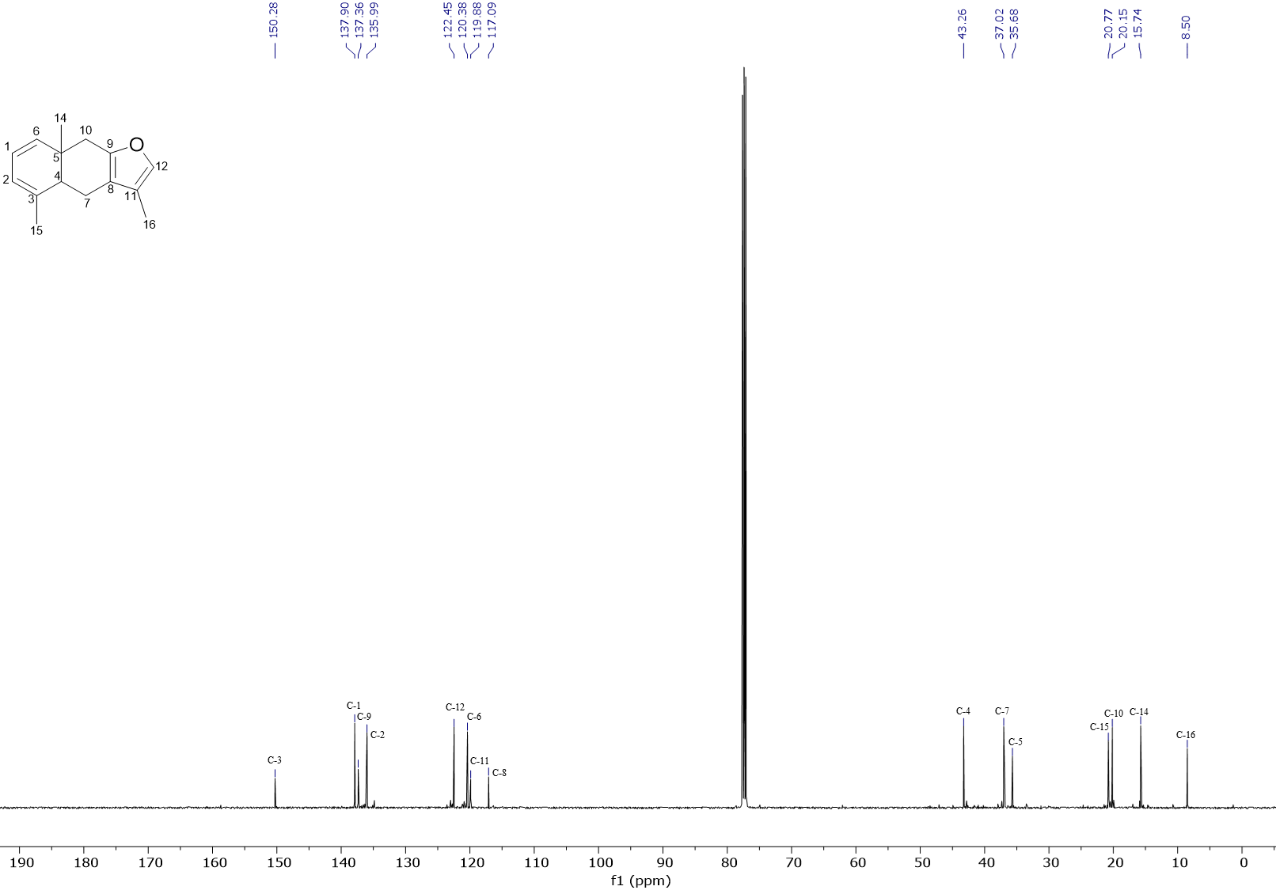
**

**Fig. S3** ^13^C NMR spectrum of furanoeudesma-1,3-diene (in CDCl_3_)


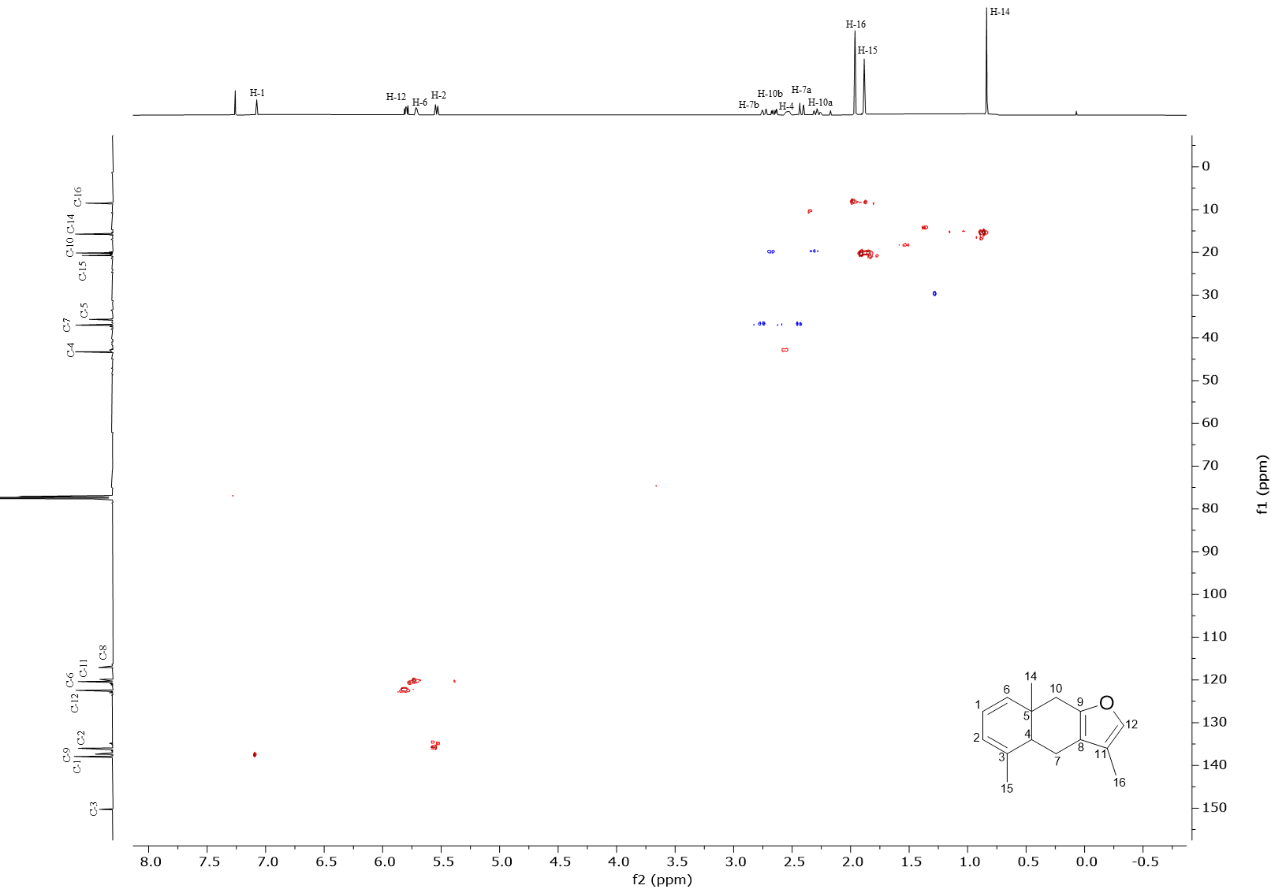


**Fig. S4** HSQC spectrum of furanoeudesma-1,3-diene (in CDCl_3_)

**2.3 HPLC-DAD quantitative analysis**

**Table S4** Quantification of the main furanosesquiterpenoids in Commiphora myrrha essential oil (EO) and fraction 2

| **Furanosesquiterpenoids** | **Concentration (g/100 g EO^a^)^b^ ± SD^c^** | **RSD %^d^** |
| --- | --- | --- |
| **EO** |  |  |
| Furanoeudesma-1,3-diene | 68.42 ± 2.36 | 3.45 |
| Isofuranodiene | 7.40 ± 0.24 | 3.27 |
| Curzerene | 18.03 ± 0.78 | 4.34 |
| **Fraction 2** |  |  |
| Furanoeudesma-1,3-diene | 69.85 ± 1.56 | 2.23 |
| Isofuranodiene | 8.03 ± 0.53 | 6.60 |
| Curzerene | 19.70 ± 0.78 | 3.95 |

^a^EO, essential oil; ^b^Average concentration (g/100 g EO) represents the concentration of each furanosesquiterpenoid and derives from two independent analyses; ^c^SD, standard deviation. ^d^RSD%, relative SD


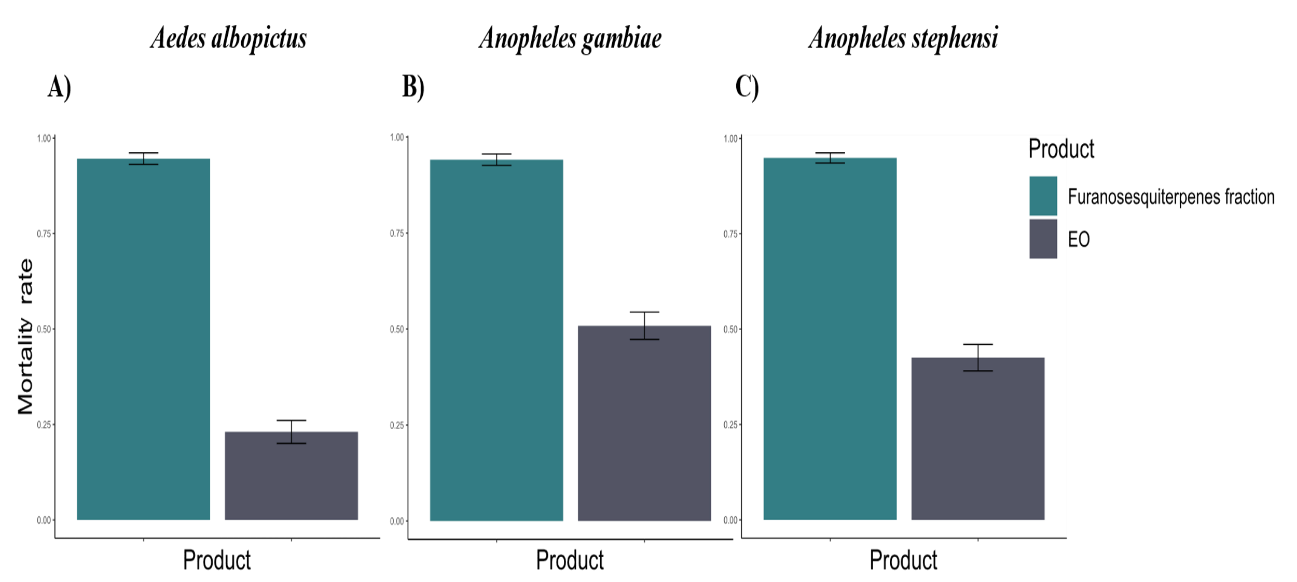


**Fig. S5** Overall species mortality rate of A) Aedes albopictus, B) Anopheles gambiae, and C) Anopheles stephensi with the essential oil (EO) and the furanosesquiterpenes fraction regardless of the tested concentrations


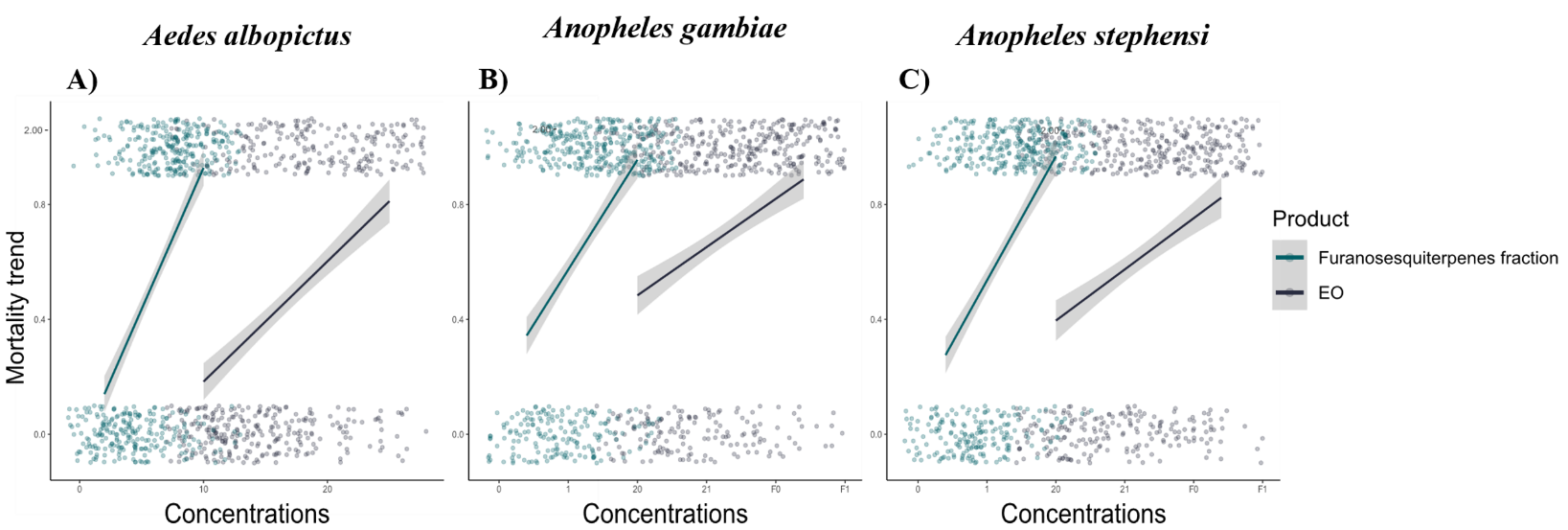


**Fig** **S6** Mortality trend of A) *Aedes albopictus*, B*) Anopheles gambiae*, and C) *Anopheles stephensi* by increasing the concentration of the essential oil (EO) and the furanosesquiterpenes fraction. Colored dots indicate the total number of tested individuals clustered around 0 when alive or around 1 when dead

**Table S5** Significantly affected features identified by volcano plot. Those characterized by a negative Log_2_ (FC) are down-accumulated, whereas those with regular font are up-accumulated. FC – fold change; Log_2_(FC) – logarithm of the FC; p.adjusted – t-test P value adjusted through FDR; LOG_10_(p) – logarithm of the adjusted t-test P value. N=5

| **Metabolites** | **FC** | **log_2_(FC)** | **p.ajusted** | **-LOG_10_(p)** |
| --- | --- | --- | --- | --- |
| Asparagine | 0.01979 | **-5.6591** | 2.34E-13 | 12.631 |
| D-Glucarate | 0.074986 | **-3.7372** | 1.36E-09 | 8.8658 |
| Sorbitol 6-phosphate | 0.12952 | **-2.9487** | 1.26E-10 | 9.901 |
| Arachidonic acid | 0.15283 | **-2.71** | 0.000286 | 3.5441 |
| 6-Phosphogluconic acid | 0.15753 | **-2.6663** | 3.84E-10 | 9.4154 |
| O-Acetylserine | 0.16043 | **-2.64** | 7.87E-11 | 10.104 |
| Methionine sulfoxide | 0.16359 | **-2.6118** | 2.91E-13 | 12.536 |
| Homocysteine | 0.16767 | **-2.5763** | 5.43E-11 | 10.265 |
| Serine | 0.19858 | **-2.3322** | 1.34E-14 | 13.873 |
| Methylmaleic acid | 0.24347 | **-2.0382** | 9.55E-08 | 7.0198 |
| Glycolic acid | 0.27199 | **-1.8784** | 0.022631 | 1.6453 |
| DL-beta-Hydroxybutyric acid | 0.29551 | **-1.7587** | 5.72E-10 | 9.2423 |
| HydroxyButyric acid | 0.29558 | **-1.7584** | 5.72E-10 | 9.2423 |
| Oleic acid | 0.30358 | **-1.7198** | 0.000916 | 3.0382 |
| Citric acid | 0.32342 | **-1.6285** | 2.51E-12 | 11.6 |
| 2-hydroxyglutaric acid | 0.34205 | **-1.5477** | 1.21E-12 | 11.917 |
| Icosanoic acid | 0.34482 | **-1.5361** | 0.00163 | 2.7879 |
| 3-Amino-2-piperidone | 0.38174 | **-1.3893** | 7.64E-10 | 9.1169 |
| Stearic acid | 0.39046 | **-1.3567** | 0.00322 | 2.4921 |
| Benzylamine | 0.40425 | **-1.3067** | 0.000293 | 3.5325 |
| Serotonin | 0.40687 | **-1.2974** | 8.01E-07 | 6.0965 |
| Galactitol | 0.41729 | **-1.2609** | 8.85E-07 | 6.0529 |
| Pipecolic acid | 0.50148 | **-0.99572** | 6.68E-05 | 4.175 |
| *N*-Acetyl aspartic acid | 0.51633 | **-0.95365** | 1.23E-08 | 7.9116 |
| 2-Oxoglutaric acid | 0.52068 | **-0.94154** | 9.66E-08 | 7.0152 |
| Threonine | 0.52501 | **-0.92957** | 2.38E-11 | 10.624 |
| Palmitic acid | 0.53216 | **-0.91008** | 0.006014 | 2.2208 |
| Glycyl-proline | 0.53299 | **-0.90783** | 3.50E-11 | 10.456 |
| Fumaric acid | 0.54011 | **-0.88866** | 2.54E-09 | 8.5947 |
| *N*-acetyl-D-mannosamine | 0.54718 | **-0.86992** | 1.22E-06 | 5.9152 |
| Glycine | 0.57855 | **-0.78948** | 8.16E-12 | 11.088 |
| Lauric acid | 0.57894 | **-0.78852** | 3.25E-05 | 4.4877 |
| R-(-)-1-Amino-2-propanol | 0.58332 | **-0.77764** | 4.29E-10 | 9.3675 |
| Malonic acid | 0.58564 | **-0.7719** | 0.000669 | 3.1749 |
| Galactosamine | 0.58795 | **-0.76624** | 3.71E-10 | 9.4309 |
| D-Xylulose | 0.60456 | **-0.72604** | 1.63E-08 | 7.7872 |
| 6-Aminohexanoic Acid | 0.60729 | **-0.71954** | 2.54E-09 | 8.5947 |
| Cytosine | 0.60757 | **-0.71889** | 0.005403 | 2.2674 |
| Sorbitol | 0.62757 | **-0.67214** | 2.54E-09 | 8.5947 |
| Malic acid | 0.64239 | **-0.63848** | 3.27E-11 | 10.486 |
| Methylmalonic acid | 0.65047 | **-0.62044** | 3.89E-05 | 4.4098 |
| Pyrogallol | 0.65099 | **-0.61928** | 1.53E-05 | 4.8161 |
| 3-Hydroxy kynurenine | 1.5098 | 0.59439 | 0.002395 | 2.6207 |
| Galactose | 1.5298 | 0.61332 | 5.38E-08 | 7.2689 |
| Heptadecanic acid | 1.5696 | 0.65043 | 0.009413 | 2.0263 |
| Oxalic acid | 1.6174 | 0.6937 | 8.19E-09 | 8.0867 |
| Phosphoenolpyruvic acid | 1.6277 | 0.70281 | 7.67E-07 | 6.1152 |
| isohexonic acid | 1.641 | 0.71458 | 2.67E-11 | 10.573 |
| Cysteine | 1.7284 | 0.7894 | 1.56E-10 | 9.8064 |
| Uridine | 1.755 | 0.8115 | 0.015015 | 1.8235 |
| 3-Amino isobutyric acid | 1.796 | 0.84475 | 6.31E-10 | 9.2002 |
| Inosine | 1.8329 | 0.87416 | 8.24E-08 | 7.084 |
| Succinic acid | 1.8352 | 0.87595 | 2.73E-10 | 9.5642 |
| Dopamine | 1.9066 | 0.93098 | 1.35E-10 | 9.8704 |
| 4-Hydroxyphenethyl alcohol | 2.0327 | 1.0234 | 5.30E-07 | 6.2758 |
| Adipic acid | 2.0419 | 1.0299 | 4.07E-06 | 5.3903 |
| Nicotinic acid | 2.2346 | 1.16 | 5.76E-09 | 8.2397 |
| Glycylglycine | 2.2351 | 1.1603 | 1.80E-08 | 7.7445 |
| Ribose | 2.2554 | 1.1734 | 3.04E-08 | 7.5175 |
| Gluconic acid | 2.2711 | 1.1834 | 2.51E-12 | 11.6 |
| Sucrose | 2.4614 | 1.2995 | 5.11E-06 | 5.2912 |
| Dopa | 2.5125 | 1.3291 | 1.19E-10 | 9.9256 |
| Ribulose 5-phosphate | 2.5206 | 1.3338 | 1.02E-08 | 7.9905 |
| Orotic acid | 2.6215 | 1.3904 | 5.90E-09 | 8.2295 |
| Putrescine | 2.6487 | 1.4053 | 8.77E-13 | 12.057 |
| *N*-acetyl-D-hexosamine | 2.7529 | 1.461 | 4.12E-12 | 11.385 |
| Cadaverine | 2.9632 | 1.5671 | 5.76E-09 | 8.2397 |
| Thymine | 3.0183 | 1.5937 | 0.001525 | 2.8167 |
| 4-HydroxyMandelic acid | 3.0331 | 1.6008 | 5.16E-12 | 11.287 |
| 3_4-Dihydrophenylacetic acid | 3.0649 | 1.6158 | 2.01E-11 | 10.697 |
| Arabitol | 3.2138 | 1.6843 | 2.48E-12 | 11.605 |
| Indole-3-acetic acid | 3.3077 | 1.7258 | 0.005007 | 2.3004 |
| homoserine | 3.6265 | 1.8586 | 1.71E-11 | 10.766 |
| *trans*-4-Hydroxy-L-proline | 4.0913 | 2.0326 | 6.62E-12 | 11.179 |
| Inositol | 4.206 | 2.0724 | 5.71E-16 | 15.244 |
| 2-Aminoethanol | 4.5746 | 2.1936 | 4.92E-10 | 9.3077 |
| GABA | 4.8609 | 2.2812 | 3.43E-14 | 13.465 |
| Uracil | 5.9975 | 2.5844 | 8.29E-11 | 10.081 |
| Urocanate | 11.838 | 3.5654 | 8.19E-09 | 8.0867 |
| 2-Deoxyinosine | 12.334 | 3.6246 | 6.95E-08 | 7.1579 |
| *N*-Acetyl glucosamine | 12.581 | 3.6532 | 6.54E-12 | 11.184 |
| 1_2 Diaminopropane | 13.592 | 3.7646 | 1.34E-14 | 13.873 |
| 1-Amino-1-cyclopentanecarboxylic acid | 17.205 | 4.1048 | 4.39E-14 | 13.357 |
| Hypoxanthine | 23.644 | 4.5634 | 2.55E-15 | 14.593 |
| Xanthine | 25.48 | 4.6713 | 1.34E-14 | 13.873 |
| 6-Hydroxynicotinic acid | 75.026 | 6.2293 | 4.13E-13 | 12.385 |
| Spermidine | 94.289 | 6.559 | 4.73E-08 | 7.3256 |

**Table S6** Metabolic pathways significantly impacted in larvae of *Aedes aegypti* treated with *Commiphora myrrha* EOs

| **Metabolic Pathways** | **Total Cmpd^a^** | **Hits^b^** | **Raw *P*** | **FDR** | **Impact** |
| --- | --- | --- | --- | --- | --- |
| Phenylalanine tyrosine and tryptophan biosynthesis | 4 | 2 | 2.58E-09 | 4.40E-09 | 1 |
| Alanine aspartate and glutamate metabolism | 23 | 8 | 5.28E-15 | 8.09E-14 | 0.80406 |
| Glycine serine and threonine metabolism | 29 | 5 | 3.49E-16 | 8.02E-15 | 0.66696 |
| Sucrose metabolism | 14 | 6 | 3.10E-08 | 4.76E-08 | 0.61392 |
| Arginine and proline metabolism | 29 | 7 | 7.08E-11 | 1.81E-10 | 0.5584 |
| Tyrosine metabolism | 33 | 6 | 3.65E-06 | 4.79E-06 | 0.48373 |
| beta-Alanine metabolism | 16 | 7 | 1.40E-09 | 2.81E-09 | 0.43322 |
| Histidine metabolism | 9 | 1 | 0.0004 | 0.0005 | 0.4 |
| Phenylalanine metabolism | 7 | 2 | 2.58E-09 | 4.40E-09 | 0.37931 |
| Glycerolipid metabolism | 12 | 3 | 3.33E-11 | 9.59E-11 | 0.37383 |
| Tryptophan metabolism | 30 | 4 | 8.85E-06 | 1.13E-05 | 0.36265 |
| Glyoxylate and dicarboxylate metabolism | 24 | 7 | 1.59E-13 | 1.22E-12 | 0.34167 |
| Fructose and mannose metabolism | 18 | 3 | 8.96E-08 | 1.33E-07 | 0.3188 |
| Cysteine and methionine metabolism | 32 | 4 | 1.41E-14 | 1.62E-13 | 0.31039 |
| Pentose phosphate pathway | 21 | 6 | 8.36E-12 | 4.27E-11 | 0.28532 |
| Citrate cycle (TCA cycle) | 20 | 6 | 4.79E-14 | 4.41E-13 | 0.2556 |
| Amino sugar and nucleotide sugar metabolism | 40 | 5 | 3.18E-11 | 9.59E-11 | 0.25503 |
| Pyrimidine metabolism | 40 | 7 | 3.16E-06 | 4.28E-06 | 0.24119 |
| Arginine biosynthesis | 12 | 9 | 5.40E-07 | 7.76E-07 | 0.22857 |
| Pentose and glucuronate interconversions | 17 | 2 | 1.18E-09 | 2.46E-09 | 0.21687 |
| Galactose metabolism | 15 | 5 | 4.24E-09 | 6.81E-09 | 0.2 |

^a^Total Cmpd: the total number of compounds in the pathway; ^b^Hits: the matched number from the uploaded data; Raw *P*: the original *P* value calculated from the enrichment analysis; FDR: the *P* value validated through the False Discovery Rate; Impact: the pathway impact value calculated from pathway topology analysis

**References**

1. Williams CM, Mander LN. Chromatography with Silver Nitrate. Tetrahedron. 2001;57(3): 425–447; <https://doi.org/10.1016/S0040-4020(00)00927-3>
